# A Systematic Review of the Mechanisms Involved in Immune Checkpoint Inhibitors Cardiotoxicity and Challenges to Improve Clinical Safety

**DOI:** 10.3389/fcell.2022.851032

**Published:** 2022-03-30

**Authors:** Nestor Rubio-Infante, Yoel Adbel Ramírez-Flores, Elena Cristina Castillo, Omar Lozano, Gerardo García-Rivas, Guillermo Torre-Amione

**Affiliations:** ^1^ Tecnologico de Monterrey, Escuela de Medicina y Ciencias de la Salud, Cátedra de Cardiología y Medicina Vascular, Monterrey, Mexico; ^2^ Tecnologico de Monterrey, Centro de Investigación Biomédica, Hospital Zambrano Hellion, TecSalud, San Pedro Garza García, Mexico; ^3^ Tecnologico de Monterrey, The Institute for Obesity Research, Monterrey, Mexico; ^4^ Tecnologico de Monterrey, Centro de Medicina Funcional, Hospital Zambrano Hellion, TecSalud, San Pedro Garza García, Mexico; ^5^ The Methodist Hospital, Cornell University, Houston, TX, United States

**Keywords:** immune checkpoint inhibitors, cardiotoxicity, CTLA-4, PD-1, myocarditis

## Abstract

Immune checkpoint inhibitors (ICIs) are monoclonal antibodies that block CTLA-4, PD-1, or PD-L1 and induce the activation of the immune system against cancer. Despite the efficacy of ICIs, which has improved the oncotherapy for patients with a variety of malignancies, several immune-related adverse events (irAEs) have been described, including those affecting the heart. Cardiac irAEs after ICI therapies, including myocarditis, can become life-threatening, and their pathogenic mechanisms remain unclear. Here, a systematic analysis was performed regarding the potential immune mechanisms underlying cardiac irAEs based on the immune adverse events induced by the ICIs: 1) recruitment of CD4^+^ and CD8^+^ T cells, 2) autoantibody-mediated cardiotoxicity, and 3) inflammatory cytokines. Furthermore, the impact of dual therapies in ICI-induced cardiac irAEs and the potential risk factors are reviewed. We propose that self-antigens released from cardiac tissues or cancer cells and the severity/advancement of cancer disease have an important role in ICI cardiotoxicity.

## 1 Introduction

T-cell-dependent immunity is mediated by T-cell receptors (TCR), co-stimulatory (e.g., CD28), and co-inhibitory molecules. The co-inhibitory molecules cytotoxic T-lymphocyte-associated antigen 4 (CTLA-4), programmed cell death 1 (PD-1), and programmed cell death-ligand 1 (PD-L1), also known as immune checkpoints (IC), act on peripheral immune tolerance regulating the response to self-proteins, inhibiting the destruction of healthy tissues, and preventing autoimmune diseases (Nirschl and Drake, 2013).

Immune checkpoint inhibitors (ICIs) are monoclonal antibodies that block IC, impeding the interaction with their respective ligands. They have been tested in preclinical models and clinical trials. Some ICIs are now approved by drug agencies, such as the European Medicines Agency (EMA) and the Food and Drug Administration (FDA) (Drugs@FDA, 2021), for use in oncotherapy to reduce the suppression of effector T cells, especially CD8^+^, improving tumor-specific immune responses (Dyck and Mills, 2017). The FDA-approved/orphan drugs ICIs include CTLA-4 inhibitors, namely, ipilimumab and tremelimumab; PD-1 inhibitors, namely, nivolumab, pembrolizumab, and cemiplimab; PD-L1 inhibitors, namely, atezolizumab, avelumab, and durvalumab (Khoja et al., 2017; Picardo et al., 2019; Vaddepally et al., 2020).

Despite the efficacy of ICIs against cancer, these therapies are accompanied by immune-related adverse events (irAEs) due to the off-target hyperactivation and dysregulation of the immune system leading to the damage of various organ systems with clinical consequences, including colitis, hepatitis, dermatitis, thyroiditis, myocarditis, or hypophysitis, which closely resemble autoimmune diseases but lack the chronicity (Haanen and Robert, 2015). In addition, some irAEs appear to be more related to specific ICIs. For example, colitis and hypophysitis are more common with anti-CTLA-4 antibodies, whereas pneumonitis and hypothyroidism are seen more frequently with anti-PD-1 therapies (Khoja et al., 2017).

Although cardiac irAEs reported in clinical trials after ICI treatments have a relatively low incidence, 3.1% in monotherapies and 5.8% in dual/combination (two different ICIs) therapies (Rubio‐Infante et al., 2021), they can be life-threatening. The hazard ratio can be increased even in monotherapies for malignant melanoma (HR = 4.3 for anti-PD-1 and 4.93 for anti-CTLA-4) (Totzeck et al., 2021). Among cardiac irAEs, as previously reported by a systematic PubMed review, myocarditis is the most frequent (51%) of cardiac irAEs, representing 0.72% of total irAEs, with an increased rate in dual ICI therapies (two-fold increased rate) (Rubio‐Infante et al., 2021). In VigiBase, WHO’s global international pharmacovigilance database, ICI-induced myocarditis ranged from 0.54% for monotherapy to 1.22% for patients treated with combination therapy with a two-fold increased rate (Wei et al., 2021). However, it has been reported that myocarditis in dual therapies could be increased up to 4.5- fold than monotherapies (the Bristol-Myers Squibb corporate safety databases (Johnson et al., 2016). The increased incidence of irAEs reported by dual therapies is likely explained by the two non-redundant co-inhibitory mechanisms. Thus, blocking these two mechanisms probably enhances synergistically T-cell autoreactivity (Thangavelu et al., 2010) within the heart.

There are three main types of heart inflammation: endocarditis (inner lining of the heart’s chambers and valves), myocarditis (heart muscle), and pericarditis (sac around the heart) that eventually can lead to deteriorating heart function ending in arrhythmias or heart failure (Tschöpe et al., 2021). Pathologically, myocarditis is characterized by myocardial infiltration of T cells and macrophages (CD4^+^, CD8^+^ T cells, and CD68^+^). The resulting inflammation leads to myocyte death and impaired heart function.

Other cardiotoxicities have been described as irAEs, including pericarditis and myocardial infarction, albeit with a lower frequency (<1%) (Pauken and Wherry, 2015). In a recent pharmacovigilance analysis, it was found that cardiac irAEs in the vigiAccess database were mainly associated with myocarditis (Rubio‐Infante et al., 2021). Recently in a metanalysis of randomized clinical trials including 32,518 patients, the ICIs therapies were associated with an increased risk of six different cardiac irAEs including myocarditis, pericardial diseases, heart failure, dyslipidemia, myocardial infarction, and cerebral arterial ischemia with a higher risk for myocarditis development (Peto OR: 4.42, 95% CI: 1.56–12.50, *p* < 0.01) (Dolladille et al., 2021). Despite, in a study by Agostinetto *et al.*, neither significant differences were found in cardiac irAEs between ICI and non-ICI groups (RR 1.14, 95% CI 0.88–1.48, *p* = 0.326), nor between dual ICI and single ICI groups (Agostinetto et al., 2021). The reasons for these discrepancies were discussed in the meta-analysis by Salem *et al.*, which after removing the trials without events in ICI and non-ICI arms, and keeping the same event data, confirmed the well-consolidated association between ICIs and myocarditis (risk ratio = 2.3–2.7) (Salem et al., 2021).

However, the mechanism of cardiac irAEs after ICIs treatment is still unknown. Knockout CTLA-4 and PD-L1 mice studies have shown different cardiomyopathies, and cardiac inflammatory infiltrates (Picardo et al., 2019). However, these infiltrates’ specificity and the possible antigens implied in this cardiac damage remain unclear.

Here, we performed a systematic literature review of the main ICI-induced irAEs and employed them to propose the potential mechanisms underlying heart damage after ICIs therapies. These mechanisms include 1) the recruitment of CD4^+^ and CD8^+^ T cells, 2) the autoantibody-mediated cardiotoxicity, and 3) the overproduction of inflammatory cytokines.

## 2 Methodology

### 2.1 Systematic Review of Mechanisms Associated With ICI-Induced irAEs

To perform the systematic review of the immune mechanisms associated with ICIs-induced irAEs, for CD4^+^ and CD8^+^ T cells recruitment in ICIs therapy, we performed a PubMed search (until December 2020) with the next set of keywords: “*X* and CD4 and CD8, not review, not clinical trial,” where *X* represents anti-CTLA-4, or ipilimumab, or tremelimumab, or anti-PD-1, or nivolumab, or pembrolizumab, or cemiplimab, or libtayo, or anti-PD-L1, or atezolizumab, or durvalumab, or avelumab.

Developing the following complete keywords: “anti-CTLA-4 and CD4 and CD8, not review, not clinical trial,” “ipilimumab and CD4 and CD8, not review, not clinical trial,” “Tremelimumab and CD4 and CD8, not review, not clinical trial,” “anti-PD-1 and CD4 and CD8, not review, not clinical trial,” “Nivolumab and CD4 and CD8 not review, not clinical trial”, “Pembrolizumab and CD4 and CD8, not review, not clinical trial,” “Cemiplimab and CD4 and CD8, not review, not clinical trial,” “Libtayo and CD4 and CD8, not review, not clinical trial,” “anti-PD-L1 and CD4 and CD8, not review, not clinical trial,” “Atezolimumab and CD4 and CD8 not review, not clinical trial,” “Durvalumab and CD4 and CD8 not review, not clinical trial,” “Avelumab and CD4 and CD8, not review, not clinical trial”. Additionally, the keywords to complete the search were added: ((*X* and CD4 and cancer [TIAB] OR *X* and CD8 and cancer [TIAB]) OR *X* and CD4 and CD8 [TIAB] and cancer NOT review [Publication type]), *X* are the same terms as described before.

To achieve the systematic review of mechanisms including the development of autoantibodies in ICI-mediated toxicity, the following set of the keywords were used: “*X* and autoantibody, not review, not clinical trial” or “((*X* and autoantibody and cancer [TIAB] NOT review [Publication type]),” where *X* represents the same terms as described before. To perform the systematic review of cytokine-mediated ICIs toxicity, the following set of the keywords were used: “*X* AND cytokines NOT review NOT clinical trial” or “((*X* and Cytokine and cancer [TIAB] NOT review [Publication type]),” where *X* represents the same terms as described before.

Titles and abstracts were screened, if the study complied with the inclusion criteria, then the full text, study details, and results were screened. The exclusion of studies from the systematic search was carried out if any of the following terms contained concomitant immunotherapies (such as dendritic cells (DC) cells, cytokines, oncolytic viruses, immunogenic cell death (ICD) inducers, chemotherapies, radiotherapies, resection, adjuvants (as pattern recognition receptors PRR agonist), vaccines, additional infections, additional antibody-based therapies (not related with CTLA-4, PD-1 or PD-L1 blockage), natural compounds, peptides, and articles not written in English. Additional exclusions included clinical trials, review articles, as well as *in vitro* or *in silico* studies, or without information of CD4^+^, CD8^+^, cytokines, or antibodies in the abstract.

## 3 Results

### 3.1 Data Retrieved, Curated, and Categorized for irAEs

After duplicate elimination, PubMed articles were analyzed, identifying 340 scientific studies containing T-cell recruitment, 135 studies containing autoantibodies development, and 662 studies containing cytokine production after ICI therapies. The systematic procedure is schematized in Supplementary Figure S1. A total of 160 studies describing irAEs were found, and the results are summarized in the following sections.

### 3.2 Anti-cancer ICI Therapies Induce T-Cell Recruitment

Several studies have described CD4^+^ and CD8^+^ T-cell recruitment after ICIs therapy. After the systematic review, we concluded that the anti-tumor effect of ICIs is related to the infiltration of both CD4^+^ and CD8^+^ T cells in tumors (Supplementary Table S1). The irAEs induced by anti-CTLA-4 in monotherapy are driven by both T cells and may be triggered by the increased ratio of CD8^+^: CD4^+^ T cells (Khan and Gerber, 2020). It seems that anti-CTLA-4 antibodies do not promote the depletion of CD4^+^ T cells Foxp3^+^ (Tregs) (Supplementary Table S1).

In anti-PD-1 monotherapy, several clinical and preclinical studies have concluded that infiltrating and circulating CD4^+^ and CD8^+^ T cells are important in the anti-tumor effect of ICIs (Supplementary Table S2), with major anti-tumor responses biased to the CD8^+^ T-cell effects. The irAEs induced by PD-1 monotherapy are elicited by both CD4^+^ and CD8^+^ T-cell populations, as suggested by their infiltration in the focal area of the irAEs. In anti-PD-L1 monotherapy, the irAEs might be linked mainly to CD4^+^ and CD8^+^ T cells since both populations have been found to increase in circulation and intratumoral sites. However, the specificity and activity have not been tested (Supplementary Table S3). Existing data in dual therapy (anti-CTLA-4 + anti-PD-1) also demonstrated the importance of CD4^+^ and CD8^+^ T-cell recruitment in the anti-tumor effect (Supplementary Table S4).

In this context, the preclinical models have highlighted the importance of the PD-1/PD-L1 axis to limit the T-cell response and, therefore, limit heart damage. In the CD8^+^ T-cell-mediated myocarditis model, PD-L1 expression on endothelial cells increases; consequently, the absence of PD-L1 exacerbates inflammation and promotes antibodies against cardiac proteins (Grabie et al., 2019). Moreover, the lack of PD-1 on this model increases the CD8^+^ response and cardiac damage (Tarrio et al., 2012). This phenomenon is also reproduced in a CD4^+^ T-cell-dependent model of autoimmune myocarditis, in which PD-1 absence enhanced cardiac damage (Tarrio et al., 2012). Preclinical models also have shown that anti-CTLA-4 imposes major boundaries on CD4^+^ T-cell phenotypes, whereas PD-1 subtly limits CD8^+^ T-cell phenotypes.

### 3.3 ICI-Induced Autoantibodies and Cardiac Damage

This systematic review highlighted the development of several autoantibodies after ICIs therapy. However, we did not study the association with the anti-tumoral effect (Supplementary Table S4). The most common irAEs in which autoantibodies have been described are found in 1) anti-CTLA-4 treatment (thyroid dysfunction), 2) anti-PD-1 treatment (myasthenia gravis/myopathy), and 3) anti-PD-L1 therapies (diabetic ketoacidosis), although with fewer reports than the former therapies.

Currently, the possible role of autoantibodies in cardiac irAEs remains unclear. One report describes that myositis related to the use of anti-PD-1 and anti-PD-L1 therapies could be a marker of subsequent myocarditis induced by these ICIs (Supplementary Table S4). Indeed, it is well-known that patients with myasthenia gravis could further develop myocarditis and myositis related to autoantibody cross-reactivity (Suzuki et al., 2017). In these reports, the autoantibodies induced by ICI therapy that could be associated with myositis and myocarditis are anti-acetylcholine receptors, anti-striated muscle antibodies, anti-mitochondrial antibodies, anti-alanyl-tRNA synthetase, anti-signal recognition particle (SRP) antibodies, and anti-3-hydroxy-3-methylglutaryl-coenzyme A reductase (Supplementary Table S4).

**FIGURE 1:**
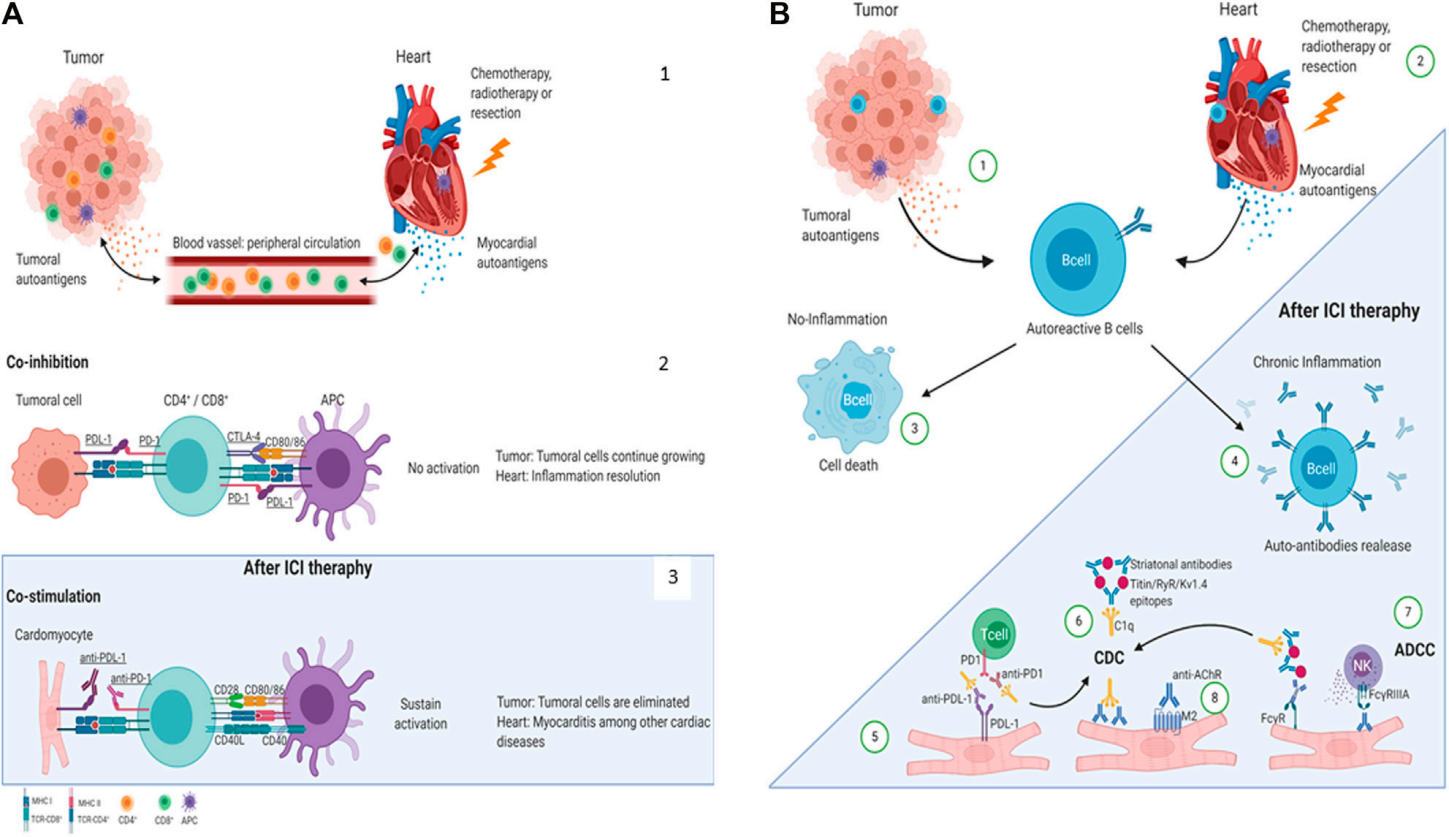
Proposed mechanisms of ICI-related cardiac irAEs. **(A)** Cardiac antigens are released by injury or stress (Nirschl and Drake, 2013). T-cell inhibition by the co-inhibitory molecules CTLA-4, PD-1, PD-L1 (Drugs@FDA, 2021). ICIs block the co-inhibitory molecules on T-cell or APC and induce anti-tumor effects (Drugs@FDA, 2021) or, in some cases, heart damage (Dyck and Mills, 2017). **(B)** Mechanisms of autoantibodies mediated cardiac irAEs after ICI therapies. Tumoral (Nirschl and Drake, 2013) and cardiac (Drugs@FDA, 2021) auto-antigens might be released by dying cells and damaged tissue in response to chemotherapy, radiotherapy, or resection and be recognized by autoreactive B cells. B cells cannot be activated in the absence of an inflammatory milieu and become non-viable. (Dyck and Mills, 2017). However, ICI therapies promote a chronic proinflammatory milieu allowing autoreactive B-cell activation to produce autoantibodies (Vaddepally et al., 2020). The PD-L1 expression has been reported in cardiomyocytes in the heart; therefore, anti-PD-L1-mediated CDC or ADCC may be directed to the heart inducing cardiac irAEs (Khoja et al., 2017). Striational antibodies might form an immune complex and CDC (Picardo et al., 2019). Finally, autoantibodies can directly recognize their antigen on the cardiomyocyte’s surface, induce ADCC (Haanen and Robert, 2015), or promote an agonist/antagonist response (Rubio‐Infante et al., 2021). AChR: acetylcholine receptor; ADCC: antibody-dependent cellular cytotoxicity; APC: antigen-presenting cell; C1q: complement component 1q; CD: Cluster of differentiationCD4+: T helper cell; CD8+: T cytotoxic cell; CDC: complement-dependent cytotoxicity; CTLA-4: cytotoxic T-lymphocyte-associated antigen 4; FcγR: Fc gamma receptors; Kv1.4: potassium voltage-gated channel; M2: muscarinic acetylcholine receptor 2; PD-1: programmed death 1; PD-L1: programmed death-ligand 1; RyR: ryanodine receptor. Created with BioRender.com.

Anti-striated muscle antibodies may cross-react with both skeletal and cardiac striated muscle antigens during myositis or myocarditis, inducing antibody-dependent cellular cytotoxicity (ADCC) (Fazal et al., 2020) through the effector functions of the Fc region of the antibodies, which is recognized by specific receptors, principally on natural killer cells, monocytes, macrophages, neutrophils, eosinophils, and dendritic cells (Wang et al., 2015). These effector immune cells induce lysis of the target cell via cytotoxic granule release, Fas signaling, and the initiation of reactive oxygen species. On the other hand, autoantibodies may induce complement-dependent cytotoxicity (CDC) activation (Duensing and Watson, 2018), ending with the target cell lysis and increasing the damage to the heart (Figure 1).

Supporting preclinical data have shown that co-inhibitory molecules are essential to prevent cardiac diseases (Grabie et al., 2019) in which antibodies are related (Nishimura et al., 2001; Okazaki et al., 2003). PD-1 deficiency causes dilated cardiomyopathy mediated by antibodies deposition on cardiac cells in mice with a Th2 bias response (Nishimura et al., 2001; Okazaki et al., 2003), which are more prompt to develop a humoral response (Busch et al., 2016). In contrast, mice with a Th1 bias (cellular immune response) developed atrial fibrillation (Fu et al., 2013). Moreover, exacerbated inflammation in preexistent inflammation stimuli, induced by the absence of co-inhibitory molecules, promotes autoantibodies related to cardiac self-antigens (Grabie et al., 2007). Furthermore, antibodies against cardiac antigens exogenously administrated in wild-type mice induced heart dysfunction (Okazaki et al., 2003). Altogether, these data highpoint the role of cardiac antibodies in promoting ICI-mediated heart disease.

### 3.4 Inflammatory Cytokine Production in ICI Toxicity

According to this systematic review, there are few reports regarding the inflammatory cytokine production in ICI toxicity (Supplementary Table S5). IFN-ƴ was the main cytokine identified in ICI toxicity. Other cytokines and chemokines such as interleukin 1β (IL-1β) and the chemokine (C-X-C motif) ligand 10 (CXCL10) were also identified. However, the evidence is not enough to determine their role in ICI cardiotoxicities (Supplementary Table S5).

During heart damage, proinflammatory cytokines and chemokines are released by cardiomyocytes, epithelial cells, and fibroblasts (Hanna and Frangogiannis, 2020; Dick and Epelman, 2016; Shirazi et al., 2017), inducing the recruitment of neutrophils and macrophages to the heart. They are key players in perpetuating inflammatory and chemoattracting factors, such as CXCL10, which induces T-cell recruitment. Binding between self-antigens and T cells induces anergy and T-cell apoptosis under normal conditions. However, when ICI therapy is administered, T-cell activity increases and worsens in the heart, inducing further injury, damage-associated molecular patterns (DAMPs) release, self-antigen liberation, and increased proinflammatory cytokines in the myocardium.

On the other hand, proinflammatory molecules may cause direct cardiac dysfunction. In this context, inflammatory molecules, such as IL-6 and C-reactive protein, increase the risk of supraventricular and ventricular cardiac arrhythmias and their complications (Kanda and Takahashi, 2004; Dick and Epelman, 2016). Moreover, immune-related myocardial inflammation could cause sick sinus and inter-atrial block (Darnell et al., 2020; Tajiri et al., 2018). Regarding the cardiac pro-arrhythmic effects caused by ICI therapy, Johnson *et al.* recently reported a case of complete heart block and cardiac arrest following initial doses of nivolumab and ipilimumab (Johnson et al., 2016).

Experimental evidence shows that cytokines directly affect cardiomyocytes altering the excitation–contraction coupling by affecting calcium handling, enabling spontaneous calcium release (Fernández-Sada et al., 2017), and facilitating the substrate to ventricular arrhythmias (Colman, 2019), (Chen et al., 2020), (Kouvas et al., 2018). For example, it has been shown that IL-1β increases calcium sparks in cardiomyocytes favoring arrhythmias associated with CaMKII oxidation and phosphorylation (Monnerat et al., 2016). On the other hand, TNF-α can modulate sarcoplasmic reticulum Ca-ATPases (SERCA2a) methylation, altering calcium handling and promoting cell dysfunction (Kao et al., 2010).

## 4 Discussion

### 4.1 Proposed Mechanisms Underlying Cardiac irAEs

The mechanisms involved in cardiac irAEs after ICI treatment remain unclear; however, here, we propose that concurrent injury or stress is required to induce cardiac irAEs (Figure 1A). Under normal conditions, T-cell responses are in homeostasis due to the actions of co-inhibitory molecules (CTLA-4, PD-1, and PD-L1) promoting inflammatory resolution. However, tumor cells often overexploit this mechanism, preventing them from being eliminated (Figure 1A2). Indeed, when ICIs are administered to patients with cancer, these cells are removed. Therefore, we hypothesize that in some cases, when a concomitant heart injury exists, the release of self-antigens promotes cardiac inflammation and damage, and due to ICI treatment, this response avoids cardiac inflammation resolution (Figures 1A3). Furthermore, we propose that self-antigens released due to chemotherapy, stress, or elimination from cancer cells can cross-react with cardiac antigens, enhancing the inflammatory signals (Figure 1B). However, these hypotheses need to be demonstrated.

Because several ICIs are administrated after the first-line treatments such as chemotherapy or radiotherapy, the injury may be triggered by these first-line treatments. Typical examples of this phenomenon are anthracyclines, including doxorubicin, daunorubicin, and epirubicin, the best-studied anti-cancer agents with cardiotoxic side effects (Dick and Epelman, 2016). For example, doxorubicin leads to the necrosis of cardiomyocytes and, therefore, the release of DAMPs, which could induce immune activation against cardiac self-antigens. The release of DAMPs may also be provoked by chemotherapy, radiotherapy, or resection of the tumor (Kouvas et al., 2018) due to the damage, necrosis, or stress in cancer (or other) cells, and these DAMPs or self-antigens could cross-react with some heart and skeletal muscle proteins.

Therefore, both preexistent cardiac injury and injury in other tissues may act as inflammation trigger signals. When the administration of ICI therapy occurs, the resolution of inflammation is blocked, promoting cardiac inflammation mediated by T cells (Nirschl and Drake, 2013), the production of cardiac antibodies (Drugs@FDA, 2021), and the impaired cardiac cell function mediated by autoantibodies and cytokines signaling (Dyck and Mills, 2017). Additionally, PD-L1, expressed by cardiac cells, might be directly recognized by ICIs, and promotes cardiac cell death by mechanisms such as the ADCC (Vaddepally et al., 2020). Recent evidence showed that T-cell infiltration is an important mechanism involved in ICI cardiotoxicity. Wei et al., 2021 described a genetic mouse model that recapitulates ICI-associated myocarditis in patients and results in premature mice death, myocardial infiltration (T cells and macrophages), and severe electrocardiogram abnormalities (Wei et al., 2021). Bocksthaler et al. (2020) showed that Troponin I (TnI)-directed autoimmune myocarditis (TnI-AM) is mediated by CD4^+^ T-cells, and the immunoproteasome is a key player in this autoimmunity. These characteristics are also presented in 2 cases of ICI-related myocarditis demonstrating the ICI-induced autoimmunity (Bockstahler et al., 2020). The study published by Michel et al. (2022) reported a murine model on which anti-PD1 therapy promotes myocardial infiltration (CD4^+^ and markedly activated CD8^+^ T cells) and impaired left ventricular (LV) function. The analogous results of impaired LV function were found in patients with metastatic melanoma receiving anti-PD1 therapy. Remarkably, they found that the blockade of TNFα preserved the LV function without attenuating the anti-cancer efficacy of anti-PD1 therapy (Michel et al., 2022). Finally, the study of Finke et al. (2021) found leukocytic infiltration (CD3^+^ and CD8^+^ cells) in cardiac biopsies from patients with ICI-associated myocarditis and the upregulation of IFN-ƴ pathway, which may indicate a possible link to the inflammasome activation and M1 macrophages activity(Fujiwara et al., 2016). Recently, a preprint article highlights the importance of CD8^+^ T cells in a genetic murine model (Pdcd1−/−Ctla4+/−) that recapitulates clinicopathologic characteristics of ICI-myocarditis. They identified the alpha-myosin as a cardiac-specific antigen; peptides from this protein induce the expansion of peripheral blood T cells from two patients with ICI-myocarditis indicating its clinical importance (Balko et al., 2022).

### 4.2 Direct Binding of anti-PD-L1 to PD-L1 Expressed in the Heart

It has been proposed that the PD1-PD-L1 pathway protects the heart and lungs from autoimmune damage (Colman, 2019) since PD-1 deficiency has been shown to induce fatal myocarditis in Murphy Roths Large (MRL) mice (Chen et al., 2020). In this scenario, the direct binding of anti-PD-L1 to the PD-L1 expressed on cardiomyocytes contributes to the failure in maintaining chronic T-cell activation and thus the loss of tolerance.

### 4.3 Challenges to Improving Clinical Safety

#### 4.3.1 The Role of Combination Therapy in the ICI Cardiotoxicity

Since 2015, when the FDA approved combination immunotherapy with ipilimumab (anti-CTLA-4) and nivolumab (anti-PD-1) (Hellmann et al., 2019), the use of dual therapies has increased due to its higher efficacy than monotherapy against several cancers, including melanoma, non-small cell lung cancer, and colorectal cancer (Larkin et al., 2019; Baas et al., 2021; Overman et al., 2018). It has been reported that dual therapy (anti-CTLA-4 and anti-PD-1/PD-L1) increased the rate of myocarditis than monotherapy (1.22% vs. 0.54%; 5.8% vs*.* 3.1%) (Wei et al., 2017; Rubio‐Infante et al., 2021). In a recent metanalysis, it was found that dual therapies have almost 50% more cardiac irAEs than monotherapies (Rubio‐Infante et al., 2021). The cardiac irAEs associated with a combination of ICIs required discontinuation of treatment in nearly 40% of patients (Varricchi et al., 2017), resulting in detrimental to cancer treatment. Moreover, in a recent systematic literature review performed in PubMed published articles, it was found that a significant number of cardiac irAEs were induced by ICIs in the first and second doses (Rubio‐Infante et al., 2021). Dual therapies improve anti-cancer effects but could increase cardiac irAEs. The explanation of the increased toxicity of dual therapies likely obeys the different signaling pathways inhibited by PD-1 and CTLA-4 molecules on T Cells (Supplementary Figure S2). T-cell activation requires two signals mediated by the interactions of TCR-MHC-Antigen (signal 1) and co-stimulatory molecules-CD28 (signal 2). On the one hand, CTLA-4 inhibits the engagement and full activation of the T-cell receptor (TCR) at priming sites (Signal 2), such as lymph nodes. On the other hand, the blockage of the PD-1/PD-L1 axis unleashed the inhibition on the signaling TCR pathway molecules (Signal 1), restoring the effector function on the inflamed peripheral tissues. Given the difference in ligands distribution, PD-L1 is expressed in peripheral tissues, while the PD-1/PD-L1 axis is associated with local tolerance (Buchbinder and Desai, 2016).

#### 4.3.2 Possible Risk Factors/Biomarkers Related to ICI Cardiotoxicity

One of the biggest challenges for cardio oncologists is to find relevant immune/cardiac biomarkers as troponin T, autoantibodies, or symptoms as myositis, which could be helpful in specific guidelines for exclusion criteria in the ICI treatments. To date, the diagnostic criteria include an electrocardiogram, biomarkers data and BNP and increased troponin (which is the basis for the diagnosis), and imaging criteria (i.e., magnetic resonance imaging) (Ederhy et al., 2021). There is a gap in the possible risk factors associated with ICI cardiac irAEs, and authors coincide in evaluating systemic autoimmune disorders before ICI treatment. For example (Menzies et al., 2017), reported that patients with preexisting autoimmune disease or melanoma treated with ipilimumab or anti-PD-1 have 20%–30% autoimmune events. Cardiovascular pathologies such as hypertension may be a risk factor. In a systematic literature review, it was found that hypertension is the leading cardiovascular risk factor for cardiac irAEs after the ICI therapies (Rubio‐Infante et al., 2021). We propose that the severity and advancement of the cancer disease could be an initial risk factor since the ICIs are administrated as second-line treatment or in advanced, unresectable, recurrent, or metastatic cancers (Rubio‐Infante et al., 2021).

Cardiac irAE mechanisms are still unclear. We have less information about possible biomarkers in ICI-induced irAEs. It has been reported that elevated cardiac troponin levels in serum are a common biomarker for myocardial injury in ICI cardiotoxicity (Sarocchi et al., 2018). Here, we propose different mechanisms related to cardiac irAEs, such as autoantibody production. Autoantibodies induced by ICI therapy include anti-acetylcholine receptors, striated muscle, mitochondria, alanyl-tRNA synthetase, SRP-B, and 3-hydroxy-3-methylglutaryl-coenzyme, these autoantibodies were found too in a recent systematic review published by (Ghosh et al., 2022), and despite that, we did not study the association of ICI-induced autoantibodies and anti-cancer efficacy, where some authors propose that the irAEs are associated with better antitumoral effect or trend for better survival (de Moel et al., 2019; Les et al., 2021). Furthermore, the recent finding by Bocksthaler et al., 2021 showed that Troponin I (TnI)-directed autoimmune myocarditis (TnI-AM) is mediated by CD4^+^ T-cells, and the immunoproteasome is a key player in this autoimmunity. They also showed 2 cases of ICI-related myocarditis associated with TnI-directed autoantibodies, demonstrating ICI-induced autoimmunity (Bockstahler et al., 2020).

## 5 Conclusion

The mechanisms involved in cardiac irAEs after ICI treatment remain unclear. However, we propose three possible mechanisms involving T-cell recruitment, autoantibodies production, and inflammatory cytokines. A cardiac antigen or a shared self-antigen in the myocardium, skeletal muscle, or tumors, may be the initial point (Grabie et al., 2019; Kanz et al., 2016). The rapid onset of myocarditis after initiating treatment with ICIs suggests the role of preexisting autoimmunity (T-memory cells), which is boosted once ICIs block PD-1 receptors (Xia et al., 2019). A high prevalence of cardiotoxicities and muscle pathologies was found to be mutually associated. We hypothesize that chemotherapy, radiotherapy, resection, previous damage, or a preexisting autoimmune pathology trigger ICI-related cardiac irAEs. In addition, studies also suggest that patients treated with CTLA-4 inhibitors have different side effect profiles than those treated with PD-1 inhibitors.

Here, we found different possible cardiac irAE mechanisms based on the literature review, which could be most likely related to T-cell activation and T cells’ recruitment to the heart, inducing myocarditis. However, the origin, specificity, autoreactivity, and activation of T cells are important challenges for oncotherapy. We found some autoantibodies which could be associated with myositis and myocarditis since they are induced by ICI therapy as anti-striated muscle antibodies and anti-acetylcholine receptors. In addition, we do not have enough evidence regarding autoantigen release in patients treated with ICIs. However, these auto-antigens could be derived from cancer cells or a previous injury, stress, or lysis of the heart or muscle tissues; nevertheless, experimental studies are needed. This review intends to be used by cardiologists and oncologists in predicting the possible appearance of cardiac irAEs and their severity.

## Data Availability

The original contributions presented in the study are included in the article/Supplementary Material, further inquiries can be directed to the corresponding authors.
