# Supplementary material for: A Systematic Review of the Mechanisms Involved in Immune Checkpoint Inhibitors Cardiotoxicity and Challenges to Improve Clinical Safety
Source: Front Cell Dev Biol. 2022 Mar 30;10:851032. doi: 10.3389/fcell.2022.851032 (PMC9006991; doi:10.3389/fcell.2022.851032)
Supplement: Supplementary file 1 [file Table1.DOCX]

| Supplementary Table 1. T-cell recruitment after anti-CTLA-4 therapy. | | | |  |
| --- | --- | --- | --- | --- |
| ICIs | **Source** | **Findings after Treatment** | **PMID** | **REF** |
| Ipilimumab | P | **^#^**High TIL score and T cells, CD8^+^ T cells associated with antitumoral response. | 32025849 | (1) |
| Ipilimumab | P | Similar density of CD4^+^, CD8^+^ and FOXP3^+^ T cells. | 29464806 | (2) |
| Ipilimumab | P | **^#^**GCM. The immune ***infiltrate in the heart*** is mostly comprised of CD4^+^ and CD8^+^ T cells and was predominant in the responding lung metastases. | 29209563 | (3) |
| Ipilimumab | P | **^#^**TILs were CD4^+^ and CD8^+^; All responders exhibited both TIL in their lymph node metastases. | 28894934 | (4) |
| Ipilimumab | P | **^&^**An increase in the numbers of clonotypes of circulating T cells, patients with irAEs exhibited greater diversity of CD4^+^ and CD8+ T cells. | 28031229 | (5) |
| Ipilimumab | P | **^#^**Improved survival was correlated with an increase in ALC and percentages of CD4^+^ and CD8^+^ T cells. | 27169993 | (6) |
| Ipilimumab | P | **^#^**Ipilimumab induced increased tumor infiltration by fully activated (CD69⁺) CD3⁺/CD4⁺ and CD3⁺/CD8⁺ T cells. | 24498358 | (7) |
| Ipilimumab | P | **^&^** Ki67^+^ EOMES^+^ CD8^+^ and Ki67^+^EOMES^+^ CD4^+^ T cells at baseline merit further testing as biomarkers associated with outcome and irAEs. | 22788688 | (8) |
| Tremelimumab | P | **^#^**Increased CD4^+^: HLA-DR^+^, PD-1^+^; CD8^+^: HLA-DR^+^, PD-1^+^, CD4^+^ICOS^+^ and CD8^+^ICOS^+^ T cells in the peripheral blood of the treated patients. An increase in tumor-infiltrating T cells. | 30688989 | (9) |
| Tremelimumab | P | **^#^**Patients with regressing lesions had an increased frequency of CD8^+^ T cells with or without a concomitant increase in CD4^+^ cells. | 19118070 | (10) |
| anti-CTLA-4 | P | **^&^** Cryptitis and glandular inflammation were observed, as well a marked increase of all T-cell subsets (CD3^+^, CD4^+^, and CD8^+^). | 18545145 | (2) |
| anti-CTLA-4, anti-PD-1 | AM | CTLA-4 imposes major boundaries on CD4^+^ T cell phenotypes, whereas PD-1 subtly limits CD8^+^ T cell phenotypes. Blockade of CTLA-4 specifically enriched T-BET^+^ TCF1^+^ CD4^+^ T cell archetype; PD-1 blockade led to enrichment of a PD-1^+^ PD-L1^int^ CD8^+^ T cell archetype. | 30926234 | (11) |
| Ipilimumab, Nivolumab | P | **^&^** Necrotizing and/or inflammatory myopathy with CD4^+^ and CD8^+^ T cells and CD68^+^ macrophages. | 31993961 | (12) |
| Ipilimumab, Nivolumab | AM | **^&^** MNC infiltrations of varying severity in several organs, including the heart, the MNC infiltration in the myocardium was comprised primarily of T cells. Increased proliferation of CD4^+^ and CD8^+^ T cells and activated and central memory T cells in the blood, spleen, and lymph nodes. | 31085720 | (13) |
| Ipilimumab, Nivolumab | P | **^$^**Lymphocyte fraction: 84% T cells with an inverted CD4^+^:CD8^+^ ratio (1:2) | 29568696 | (14) |
| Ipilimumab, Nivolumab | P | **^$^**Lesioned skin with lymphocytic infiltration predominantly positive for CD8^+^, contrasting with those for CD4^+^ T cells. | 29515387 | (15) |
| Ipilimumab, Nivolumab | P | **^$^**Large numbers of CD8^+^ T cells than CD4^+^ T cells and CD20^+^ B cells in the liver. | 29403081 | (16) |
| Anti-CTLA-4, anti-PD1 | P | **^&^**CD4^+^ and CD8^+^ memory T cell subsets play an important role in response to anti-CTLA-4 and are potential biomarker candidates. | 29510697 | (17) |
| Ipilimumab, Tremelimumab | P | Both treatments increase intra-tumoral CD4^+^ and CD8^+^ cells' infiltration without significantly changing or depleting FOXP3^+^ cells within the tumor microenvironment. | 30054281 | (18) |
| Pembrolizumab, Atezolizumab | P | **^$^**Confluent necrosis and eosinophilic or plasma cell infiltration; *in situ,* the ratio of CD8^+^: CD4^+^ T cells was 12.2 ± 5.1, higher than in autoimmune hepatitis. | 31550390 | (19) |
| anti-CTLA-4, anti-PD-1, anti-CTLA-4 + anti-PD-L1 | AM | **^#^**Increased intra-tumoral CD8^+^ and CD4^+^ T cells and reduced FOXP3^+^ Treg cells; Increased expression levels of the proinflammatory Th1/M1-related cytokines IFN-γ, IL-1α, IL-2, and IL-12. | 31412307 | (20) |
| anti-CTLA-4 + anti-PD-1 | P | Anti-PD-1 predominantly induces the expansion of specific tumor-infiltrating exhausted-like CD8 T cell subsets; anti-CTLA-4 induces the expansion of an ICOS^+^ Th1-like CD4^+^ effector population in addition to engaging specific subsets of exhausted-like CD8^+^ T cells. | 28803728 | (21) |
| Ipilimumab + Nivolumab | AM | Increase in the percentage of CD8^+^ T cells; IFN-γ and IL-6 increased in the plasma; Tumor-infiltrating T cells secreted more IFN-γ towards *ex vivo* stimulation. | 32331230 | (22) |
| anti-CTLA-4 + anti-PD-1 | AM | **^#^**Prolonged survival; Infiltration of CD8^+^ and CD4^+^ T cells into tumors | 31796506 | (23) |
| anti-CTLA-4 + anti-PD-1 | AM | Exhausted CD8^+^ T cells expand in frequency following anti-PD-1 therapy; Combined therapy induces the expands of activated terminally differentiated effector CD8^+^ T cells and increased frequency of Th1-like CD4^+^ effector T cells. | 31636208 | (24) |
| Ipilimumab + Nivolumab | AM | Immunotherapy-activated CD8^+^ T cells promote tumor cell lipid peroxidation and sensitize tumors to ferroptosis | 31043744 | (25) |
| Ipilimumab + Nivolumab | P | **^#^**The disappearance of all malignant cells; Renal mass revealed strong positivity for CD4^+^ and CD8^+^ T cells. | 32257760 | (26) |
| Tremelimumab + durvalumab | P | **^&^**Muscle fiber atrophy with a mixed CD8^+^ and CD4^+^ T cell infiltrate, indicative of inflammatory myopathy. | 28716137 | (27) |
| Data obtained from 30 retrieved. GCM: Giant cell myocarditis; TIL: Tumor-infiltrating lymphocytes; ALC: absolute circulating lymphocyte counts; MNC: Mononuclear cell; Th1: T helper type 1 TIL: tumor-infiltrating lymphocytes. # Antitumoral responses mediated by both CD4^+^ and CD8^+^ T cells (5), ^&^ irAEs mediated by both CD4^+^ and CD8^+^ T cells, ^$^ irAEs mediated by CD8^+^ T cells. | | | |  |

1. Mastracci L, Fontana V, Queirolo P, Carosio R, Grillo F, Morabito A, et al. Response to ipilimumab therapy in metastatic melanoma patients: potential relevance of CTLA-4+ tumor infiltrating lymphocytes and their in situ localization. Cancer Immunol Immunother CII. 2020 Apr;69(4):653–62.

2. Adler BL, Pezhouh MK, Kim A, Luan L, Zhu Q, Gani F, et al. Histopathological and immunophenotypic features of ipilimumab-associated colitis compared to ulcerative colitis. J Intern Med. 2018 Jun;283(6):568–77.

3. Reuben A, Petaccia de Macedo M, McQuade J, Joon A, Ren Z, Calderone T, et al. Comparative immunologic characterization of autoimmune giant cell myocarditis with ipilimumab. Oncoimmunology. 2017;6(12):e1361097.

4. Diem S, Hasan Ali O, Ackermann CJ, Bomze D, Koelzer VH, Jochum W, et al. Tumor infiltrating lymphocytes in lymph node metastases of stage III melanoma correspond to response and survival in nine patients treated with ipilimumab at the time of stage IV disease. Cancer Immunol Immunother CII. 2018 Jan;67(1):39–45.

5. Oh DY, Cham J, Zhang L, Fong G, Kwek SS, Klinger M, et al. Immune Toxicities Elicted by CTLA-4 Blockade in Cancer Patients Are Associated with Early Diversification of the T-cell Repertoire. Cancer Res. 2017 Mar 15;77(6):1322–30.

6. Martens A, Wistuba-Hamprecht K, Yuan J, Postow MA, Wong P, Capone M, et al. Increases in Absolute Lymphocytes and Circulating CD4+ and CD8+ T Cells Are Associated with Positive Clinical Outcome of Melanoma Patients Treated with Ipilimumab. Clin Cancer Res Off J Am Assoc Cancer Res. 2016 Oct 1;22(19):4848–58.

7. Tarhini AA, Edington H, Butterfield LH, Lin Y, Shuai Y, Tawbi H, et al. Immune monitoring of the circulation and the tumor microenvironment in patients with regionally advanced melanoma receiving neoadjuvant ipilimumab. PloS One. 2014;9(2):e87705.

8. Wang W, Yu D, Sarnaik AA, Yu B, Hall M, Morelli D, et al. Biomarkers on melanoma patient T cells associated with ipilimumab treatment. J Transl Med. 2012 Jul 12;10:146.

9. Agdashian D, ElGindi M, Xie C, Sandhu M, Pratt D, Kleiner DE, et al. The effect of anti-CTLA4 treatment on peripheral and intra-tumoral T cells in patients with hepatocellular carcinoma. Cancer Immunol Immunother CII. 2019 Apr;68(4):599–608.

10. Ribas A, Comin-Anduix B, Economou JS, Donahue TR, de la Rocha P, Morris LF, et al. Intratumoral immune cell infiltrates, FoxP3, and indoleamine 2,3-dioxygenase in patients with melanoma undergoing CTLA4 blockade. Clin Cancer Res Off J Am Assoc Cancer Res. 2009 Jan 1;15(1):390–9.

11. Wei SC, Sharma R, Anang N-AAS, Levine JH, Zhao Y, Mancuso JJ, et al. Negative Co-stimulation Constrains T Cell Differentiation by Imposing Boundaries on Possible Cell States. Immunity. 2019 Apr 16;50(4):1084-1098.e10.

12. Vermeulen L, Depuydt CE, Weckx P, Bechter O, Van Damme P, Thal DR, et al. Myositis as a neuromuscular complication of immune checkpoint inhibitors. Acta Neurol Belg. 2020 Apr;120(2):355–64.

13. Ji C, Roy MD, Golas J, Vitsky A, Ram S, Kumpf SW, et al. Myocarditis in Cynomolgus Monkeys Following Treatment with Immune Checkpoint Inhibitors. Clin Cancer Res Off J Am Assoc Cancer Res. 2019 Aug 1;25(15):4735–48.

14. Meyers DE, Hill WF, Suo A, Jimenez-Zepeda V, Cheng T, Nixon NA. Aplastic anemia secondary to nivolumab and ipilimumab in a patient with metastatic melanoma: a case report. Exp Hematol Oncol. 2018;7:6.

15. Utsunomiya A, Oyama N, Iino S, Baba N, Chino T, Utsunomiya N, et al. A Case of Erythema Multiforme Major Developed after Sequential Use of Two Immune Checkpoint Inhibitors, Nivolumab and Ipilimumab, for Advanced Melanoma: Possible Implication of Synergistic and/or Complementary Immunomodulatory Effects. Case Rep Dermatol. 2018 Apr;10(1):1–6.

16. Zen Y, Yeh MM. Hepatotoxicity of immune checkpoint inhibitors: a histology study of seven cases in comparison with autoimmune hepatitis and idiosyncratic drug-induced liver injury. Mod Pathol Off J U S Can Acad Pathol Inc. 2018 Jun;31(6):965–73.

17. Subrahmanyam PB, Dong Z, Gusenleitner D, Giobbie-Hurder A, Severgnini M, Zhou J, et al. Distinct predictive biomarker candidates for response to anti-CTLA-4 and anti-PD-1 immunotherapy in melanoma patients. J Immunother Cancer. 2018 Mar 6;6(1):18.

18. Sharma A, Subudhi SK, Blando J, Scutti J, Vence L, Wargo J, et al. Anti-CTLA-4 Immunotherapy Does Not Deplete FOXP3+ Regulatory T Cells (Tregs) in Human Cancers. Clin Cancer Res Off J Am Assoc Cancer Res. 2019 Feb 15;25(4):1233–8.

19. Zen Y, Chen Y-Y, Jeng Y-M, Tsai H-W, Yeh MM. Immune-related adverse reactions in the hepatobiliary system: second-generation check-point inhibitors highlight diverse histological changes. Histopathology. 2020 Feb;76(3):470–80.

20. Fiegle E, Doleschel D, Koletnik S, Rix A, Weiskirchen R, Borkham-Kamphorst E, et al. Dual CTLA-4 and PD-L1 Blockade Inhibits Tumor Growth and Liver Metastasis in a Highly Aggressive Orthotopic Mouse Model of Colon Cancer. Neoplasia N Y N. 2019 Sep;21(9):932–44.

21. Wei SC, Levine JH, Cogdill AP, Zhao Y, Anang N-AAS, Andrews MC, et al. Distinct Cellular Mechanisms Underlie Anti-CTLA-4 and Anti-PD-1 Checkpoint Blockade. Cell. 2017 Sep 7;170(6):1120-1133.e17.

22. Liu WN, Fong SY, Tan WWS, Tan SY, Liu M, Cheng JY, et al. Establishment and Characterization of Humanized Mouse NPC-PDX Model for Testing Immunotherapy. Cancers. 2020 Apr 22;12(4):E1025.

23. Sun T, Zhang W, Li Y, Jin Z, Du Y, Tian J, et al. Combination Immunotherapy with Cytotoxic T-Lymphocyte-Associated Antigen-4 and Programmed Death Protein-1 Inhibitors Prevents Postoperative Breast Tumor Recurrence and Metastasis. Mol Cancer Ther. 2020 Mar;19(3):802–11.

24. Wei SC, Anang N-AAS, Sharma R, Andrews MC, Reuben A, Levine JH, et al. Combination anti-CTLA-4 plus anti-PD-1 checkpoint blockade utilizes cellular mechanisms partially distinct from monotherapies. Proc Natl Acad Sci U S A. 2019 Nov 5;116(45):22699–709.

25. Wang W, Green M, Choi JE, Gijón M, Kennedy PD, Johnson JK, et al. CD8+ T cells regulate tumour ferroptosis during cancer immunotherapy. Nature. 2019 May;569(7755):270–4.

26. Okada T, Hamamoto S, Etani T, Naiki T, Sue Y, Banno R, et al. Complete response of renal cell carcinoma with an inferior vena cava tumor thrombus and lung metastases after treatment with nivolumab plus ipilimumab. Int Cancer Conf J. 2020 Apr;9(2):88–91.

27. John S, Antonia SJ, Rose TA, Seifert RP, Centeno BA, Wagner AS, et al. Progressive hypoventilation due to mixed CD8+ and CD4+ lymphocytic polymyositis following tremelimumab - durvalumab treatment. J Immunother Cancer. 2017 Jul 18;5(1):54.
