# Supplementary material for: A Systematic Review of the Mechanisms Involved in Immune Checkpoint Inhibitors Cardiotoxicity and Challenges to Improve Clinical Safety
Source: Front Cell Dev Biol. 2022 Mar 30;10:851032. doi: 10.3389/fcell.2022.851032 (PMC9006991; doi:10.3389/fcell.2022.851032)
Supplement: Supplementary file 3 [file Table2.DOCX]

| Supplementary Table 2. Mechanisms of anti-PD-1 T cell recruitment | | | |  |
| --- | --- | --- | --- | --- |
| ICIs | **Source** | **Findings After Treatment** | **PMID** | **REF** |
| anti-PD-1 | P | **^#^**Increased intratumoral CD4^+^ and CD8^+^ T cell clonality in responders to Anti-PD-1 Therapy. | 32411589 | (1) |
| anti-PD-1 | P | **^&^**PE. The frequency of both CD4^+^ and CD8^+^ T cells increased. | 31639551 | (2) |
| anti-PD-1 | P | **^+^**Responders to PD-1/PD-L1 pathway blockade showed a trend of high baseline frequency of intratumoral PD-1 CD8 T cells | 31295151 | (3) |
| anti-PD-1 | P | **^&^**Pulmonary nodule demonstrated a granulomatous, CD4^+^ T-cell infiltrate, correlating with increased CD4^+^ and CD8^+^ naïve memory cells in the peripheral blood. | 31088979 | (4) |
| anti-PD-1 | P | **^+^**The patients in the clinical benefit group with PD-1 blockade showed a higher number of CD3+, CD8+ T cells and a higher CD8+/CD3+ T cell ratio (p = 0.003, p = 0.001, and p = 0.042) | 30297881 | (5) |
| anti-PD-1 | P | **^+^**The proportion of circulating T cells remained stable, with decreasing trends of CD4^+^ T cell and increased in CD8^+^ T memory cells. | 29290787 | (6) |
| anti-PD-1 | P | The bronchoalveolar lavage differential cell count showed 32% lymphocytes with an increased CD4^+^: CD8^+^ ratio. Bronchial biopsies revealed noncaseating epithelioid granulomas without malignant cells. | 27291635 | (7) |
| anti-PD-1 | P | **^#^**Revitalizes circulating Tem and Tcm of CD4^+^ and CD8^+^ T cells and induces a shift to a Th1 phenotype. | 30066977 | (8) |
| Nivolumab | P | **^+^**Clinical benefits correlate with reduced expression of SLC3A2 and increased IFN-γ and CD8 transcripts in blood. | 31043744 | (9) |
| Nivolumab | P | **^&^**Marked portal infiltration, including eosinophils and CD4^+^ and CD8^+^ T cells. | 30799364 | (10) |
| Nivolumab | P | Increased proportion of circulating CD8^+^, with increased granzyme B expression and a subpopulation with a near-significant increase in IFN-γ expression. | 32183719 | (11) |
| Nivolumab | P | **^#^**Patients characterized by longer OS had higher levels of CD4^+^ and CD8^+^ T cells, but lower levels of NK cells at baseline. | 32117275 | (12) |
| Nivolumab | P | **^&^**Renal biopsy pathologically revealed tubulointerstitial inflammation with strong infiltration of only T cells that were CD4^+^ and CD8^+^. | 32110225 | (13) |
| Nivolumab | P | Circulating CD4^+^: CD8^+^ ratio remained unchanged during therapy (mean -0.12 in 8 weeks). The respective percentage values of circulating T cells also remained stable. | 31910171 | (14) |
| Nivolumab, Pembrolizumab, Atezolizumab | P | High percentage of PD1^+^CD4^+^ T cells relative to the total CD4^+^ T cells count associated with a longer progression-free survival. | 31810958 | (15) |
| Nivolumab | P | **^$^**CD8^+^ T cells mainly infiltrate in the endomysium with a small number of CD4^+^ T cells. | 31142711 | (16) |
| Nivolumab | P | **^&^**Cellular infiltration in myocardial biopsy: CD4^+^, CD8^+^, CD20^-^ and PD-1^-^, interstitial edema, and myocardial necrosis. PD-L1 was predominantly expressed on the surface of the damaged myocardium. | 29801747 | (17) |
| Nivolumab | P | **^{^**Among infiltrating immune cells, T cells' aggregation was more extensive than that of B cells, with CD4^+^ outnumbering CD8^+^ T cells, consistent with the relative numbers of these cells on circulation. | 29623482 | (18) |
| Nivolumab | P | **^#^**Histological evaluation of the metastatic liver of one case after regression revealed fibrotic tissue containing infiltrated lymphocytes positive for CD4 or CD8 but no viable tumor cells, suggestive of a durable immune reaction even after a complete pathological response. | 27987588 | (19) |
| Nivolumab | P | **^&^**Endomyocardial biopsy showed acute moderate diffuse cellular rejection with a predominant population of CD8^+^ and CD4^+^ infiltrating T cells. A high frequency of proliferating and activated CD8^+^ circulating T cells expressing PD-1 compared to normal control. There was no difference in the activation and proliferation of CD4+ T cells compared to normal control. | 27771741 | (20) |
| Nivolumab | P, AM | An early increase in circulating Th9 cell counts was associated with an improved clinical response. | 28123885 | (21) |
| Pembrolizumab | P | **^$^**An inflammatory infiltrate, mainly of CD8^+^ T cells over CD4^+^. | 31748755 | (22) |
| Pembrolizumab | P | **^+^**In patients with NSCLC treated with PD-1 blockade with long-term follow up, TMB, PD-L1, and CD8 were each associated with benefit from PD-1 blockade | 31113840 | (23) |
| Pembrolizumab | P | There was no significant association of CD8^+^ cell density with clinical response. | 30285852 | (24) |
| Nivolumab | AM | **^&^**The dilated left ventricle was associated with an infiltration of CD4^+^ and CD8^+^ T cells into the myocardium. | 32244307 | (25) |
| anti-PD-1 | AM | **^+^**Inhibited colon cancer cell metastasis, the depletion of CD8^+^ T cells suppressed the activity of anti-PD-1 antibodies. | 31695411 | (26) |
| anti-PD-1 | AM | **^+^**The strong anti-tumor response correlated with increased IFN-γ-secreting, tumor-specific CD8^+^ T cells, but not with CD4^+^ Tregs in tumor tissue. | 31402780 | (27) |
| anti-PD-1 | AM | **^#^**Slightly enriched CD8^+^ T cells and CD4^+^ T cells; however, the immune cells' infiltration was negligible. | 30117062 | (28) |
| anti-PD-1 | AM | **^#^**PD-1 blockade therapy in tumor models requires CD4^+^ and CD8^+^ T cells and costimulation mediated by dendritic cells and macrophages. | 27589875 | (29) |
| Ipilimumab, Nivolumab, or Pembrolizumab | P | **^{^**The inflammatory infiltrate consisted of T lymphocytes (CD3+ ) with a predominance of CD4+ over CD8+ cells; | 28188628 | (30) |
| Ipilimumab + Nivolumab | P | **^{^**Predominance of CD4 > CD8 and CD68 macrophages, with occasional CD20 B cells in myocardium. | 32062109 | (31) |
| Nivolumab, Pembrolizumab Durvalumab, Ipilimumab +Nivolumab | P | **^$^**Endomysial inflammation, consisting mainly of CD68^+^ cells and CD8^+^ cells expressing PD-1. | 30089619 | (32) |
| anti-PD-1,  anti-PD-L1 | P | **^+^**Th9 cells could contribute to anti-tumor immunity by enhancing the recruitment and activation of mast cells, NK cells, CD8^+^ T cells, and dendritic cells in the tumor microenvironment. | 31776089 | (33) |
|  |  |  |  |  |

| Nivolumab | P | ^#^CD8 + and CD4 + T-cell activation and CD8 + T-cell memory formation were significantly related to an improved prognosis. | 33140187 | (34) |
| --- | --- | --- | --- | --- |
| anti-PD-1 | P | ^#^Lung metastatic lesions size were decreased after exposure to six cycles of toripalimab. The biopsy samples obtained after toripalimab treatment showed significant intratumoral CD4+ and CD8+ T‐cell infiltration. | 32994998 | (35) |
| anti-PD-1 | AM | ^#^Anti-PD-1 therapy could prevent up to 46% of liver tumors and induces the infiltration of CD4+, CD8+ T cells into the liver parenchyma. | 32839204 | (36) |
| Pembrolizumab | P | **^$^**The progression of the primary lesion and ileocecal lymph node metastasis were confirmed after therapy. Patient was diagnosed with hepatitis as an irAE. Infiltrating cells were predominantly CD3-positive (CD3+) and CD8+, and few CD20+ and CD4+ lymphocytes. | 33250701 | (37) |
| Nivolumab | P | **^&^**A marked portal infiltration, including eosinophils and CD4+ and CD8+ T lymphocytes, suggesting nivolumab-related cholangitis accompanied by adverse events (irAE). | 30799364 | (38) |

| Data obtained from 33 retrieve papers. PE: Pleural effusion; P: Patients; AM: animal model; TIM-3 Superficial perivascular lymphocytic dermatitis. ^#^ Antitumoral responses mediated by both TCD4+ and TCD8+ cells (8), ^+^ Antitumoral responses mediated by TCD8+ cells (8), ^&^ irAEs mediated by both TCD4+ and TCD8+ cells (8), ^$^ irAEs mediated by TCD8+ cells (4), ^{^ irAEs mediated by TCD4+ cells (3). |  |
| --- | --- |

1. Zhigalova EA, Izosimova AI, Yuzhakova DV, Volchkova LN, Shagina IA, Turchaninova MA, et al. RNA-Seq-Based TCR Profiling Reveals Persistently Increased Intratumoral Clonality in Responders to Anti-PD-1 Therapy. Front Oncol. 2020;10:385.

2. Ikematsu Y, Tanaka K, Yanagihara T, Liu R, Inoue H, Yoneshima Y, et al. Immune checkpoint protein and cytokine expression by T lymphocytes in pleural effusion of cancer patients receiving anti-PD-1 therapy. Lung Cancer Amst Neth. 2019 Dec;138:58–64.

3. Macek Jilkova Z, Aspord C, Kurma K, Granon A, Sengel C, Sturm N, et al. Immunologic Features of Patients With Advanced Hepatocellular Carcinoma Before and During Sorafenib or Anti-programmed Death-1/Programmed Death-L1 Treatment. Clin Transl Gastroenterol. 2019 Jul;10(7):e00058.

4. Nabel CS, Severgnini M, Hung YP, Cunningham-Bussel A, Gjini E, Kleinsteuber K, et al. Anti-PD-1 Immunotherapy-Induced Flare of a Known Underlying Relapsing Vasculitis Mimicking Recurrent Cancer. The Oncologist. 2019 Aug;24(8):1013–21.

5. Kim H, Kwon HJ, Han YB, Park SY, Kim ES, Kim SH, et al. Increased CD3+ T cells with a low FOXP3+/CD8+ T cell ratio can predict anti-PD-1 therapeutic response in non-small cell lung cancer patients. Mod Pathol Off J U S Can Acad Pathol Inc. 2019 Mar;32(3):367–75.

6. Jiang C, Cai X, Zhang H, Xia X, Zhang B, Xia L. Activity and Immune Correlates of a Programmed Death-1 Blockade Antibody in the treatment of Refractory Solid Tumors. J Cancer. 2018;9(1):205–12.

7. Montaudié H, Pradelli J, Passeron T, Lacour J-P, Leroy S. Pulmonary sarcoid-like granulomatosis induced by nivolumab. Br J Dermatol. 2017 Apr;176(4):1060–3.

8. Yamaguchi K, Mishima K, Ohmura H, Hanamura F, Ito M, Nakano M, et al. Activation of central/effector memory T cells and T-helper 1 polarization in malignant melanoma patients treated with anti-programmed death-1 antibody. Cancer Sci. 2018 Oct;109(10):3032–42.

9. Wang W, Green M, Choi JE, Gijón M, Kennedy PD, Johnson JK, et al. CD8+ T cells regulate tumour ferroptosis during cancer immunotherapy. Nature. 2019 May;569(7755):270–4.

10. Sawada K, Shonaka T, Nishikawa Y, Hasegawa K, Hayashi H, Hasebe T, et al. Successful Treatment of Nivolumab-related Cholangitis with Prednisolone: A Case Report and Review of the Literature. Intern Med Tokyo Jpn. 2019 Jun 15;58(12):1747–52.

11. Xiong Y, Neskey DM, Horton JD, Paulos CM, Knochelmann HM, Armeson KE, et al. Immunological effects of nivolumab immunotherapy in patients with oral cavity squamous cell carcinoma. BMC Cancer. 2020 Mar 17;20(1):229.

12. Ottonello S, Genova C, Cossu I, Fontana V, Rijavec E, Rossi G, et al. Association Between Response to Nivolumab Treatment and Peripheral Blood Lymphocyte Subsets in Patients With Non-small Cell Lung Cancer. Front Immunol. 2020;11:125.

13. Okawa S, Fujiwara K, Shimonishi A, Matsuura H, Ozeki T, Nishimura J, et al. Rapidly Progressive Acute Kidney Injury Associated with Nivolumab Treatment. Case Rep Oncol. 2020 Apr;13(1):85–90.

14. Bersanelli M, Gnetti L, Vaglio A, Sverzellati N, Campanini N, Incerti M, et al. Correlations between tumor-infiltrating and circulating lymphocyte subpopulations in advanced renal cancer patients treated with nivolumab. Acta Bio-Medica Atenei Parm. 2019 Dec 23;90(4):468–74.

15. Inomata M, Kado T, Okazawa S, Imanishi S, Taka C, Kambara K, et al. Peripheral PD1-positive CD4 T-Lymphocyte Count Can Predict Progression-free Survival in Patients With Non-small Cell Lung Cancer Receiving Immune Checkpoint Inhibitor. Anticancer Res. 2019 Dec;39(12):6887–93.

16. Sawai T, Hosokawa T, Shigekiyo T, Ogawa S, Sano E, Arawaka S. [An autopsy case of nivolumab-induced myasthenia gravis and myositis]. Rinsho Shinkeigaku. 2019 Jun 22;59(6):360–4.

17. Yamaguchi S, Morimoto R, Okumura T, Yamashita Y, Haga T, Kuwayama T, et al. Late-Onset Fulminant Myocarditis With Immune Checkpoint Inhibitor Nivolumab. Can J Cardiol. 2018 Jun;34(6):812.e1-812.e3.

18. Nakatani Y, Kawakami H, Ichikawa M, Yamamoto S, Otsuka Y, Mashiko A, et al. Nivolumab-induced acute granulomatous tubulointerstitial nephritis in a patient with gastric cancer. Invest New Drugs. 2018 Aug;36(4):726–31.

19. Tanizaki J, Hayashi H, Kimura M, Tanaka K, Takeda M, Shimizu S, et al. Report of two cases of pseudoprogression in patients with non-small cell lung cancer treated with nivolumab-including histological analysis of one case after tumor regression. Lung Cancer Amst Neth. 2016 Dec;102:44–8.

20. Owonikoko TK, Kumar M, Yang S, Kamphorst AO, Pillai RN, Akondy R, et al. Cardiac allograft rejection as a complication of PD-1 checkpoint blockade for cancer immunotherapy: a case report. Cancer Immunol Immunother CII. 2017 Jan;66(1):45–50.

21. Nonomura Y, Otsuka A, Nakashima C, Seidel JA, Kitoh A, Dainichi T, et al. Peripheral blood Th9 cells are a possible pharmacodynamic biomarker of nivolumab treatment efficacy in metastatic melanoma patients. Oncoimmunology. 2016;5(12):e1248327.

22. Gaffuri P, Espeli V, Fulciniti F, Paone G, Bergmann M. Immune-related acute and lymphocytic gastritis in a patient with metastatic melanoma treated with pembrolizumab immunotherapy. Pathologica. 2019 Sep;111(3):92–7.

23. Hu-Lieskovan S, Lisberg A, Zaretsky JM, Grogan TR, Rizvi H, Wells DK, et al. Tumor Characteristics Associated with Benefit from Pembrolizumab in Advanced Non-Small Cell Lung Cancer. Clin Cancer Res Off J Am Assoc Cancer Res. 2019 Aug 15;25(16):5061–8.

24. Giraldo NA, Nguyen P, Engle EL, Kaunitz GJ, Cottrell TR, Berry S, et al. Multidimensional, quantitative assessment of PD-1/PD-L1 expression in patients with Merkel cell carcinoma and association with response to pembrolizumab. J Immunother Cancer. 2018 Oct 1;6(1):99.

25. Tay WT, Fang Y-H, Beh ST, Liu Y-W, Hsu L-W, Yen C-J, et al. Programmed Cell Death-1: Programmed Cell Death-Ligand 1 Interaction Protects Human Cardiomyocytes Against T-Cell Mediated Inflammation and Apoptosis Response In Vitro. Int J Mol Sci. 2020 Mar 31;21(7):E2399.

26. Gao CE, Zhang M, Song Q, Dong J. PD-1 inhibitors dependent CD8+ T cells inhibit mouse colon cancer cell metastasis. OncoTargets Ther. 2019;12:6961–71.

27. Wang J, Fei K, Jing H, Wu Z, Wu W, Zhou S, et al. Durable blockade of PD-1 signaling links preclinical efficacy of sintilimab to its clinical benefit. mAbs. 2019 Dec;11(8):1443–51.

28. Tomita M, Yasui H, Higashikawa K, Nakajima K, Takakura H, Shiga T, et al. Anti PD-1 treatment increases [18F]FDG uptake by cancer cells in a mouse B16F10 melanoma model. EJNMMI Res. 2018 Aug 16;8(1):82.

29. Homet Moreno B, Zaretsky JM, Garcia-Diaz A, Tsoi J, Parisi G, Robert L, et al. Response to Programmed Cell Death-1 Blockade in a Murine Melanoma Syngeneic Model Requires Costimulation, CD4, and CD8 T Cells. Cancer Immunol Res. 2016 Oct;4(10):845–57.

30. Perret RE, Josselin N, Knol A-C, Khammari A, Cassecuel J, Peuvrel L, et al. Histopathological aspects of cutaneous erythematous-papular eruptions induced by immune checkpoint inhibitors for the treatment of metastatic melanoma. Int J Dermatol. 2017 May;56(5):527–33.

31. Hardy T, Yin M, Chavez JA, Ivanov I, Chen W, Nadasdy T, et al. Acute fatal myocarditis after a single dose of anti-PD-1 immunotherapy, autopsy findings: a case report. Cardiovasc Pathol Off J Soc Cardiovasc Pathol. 2020 Jun;46:107202.

32. Touat M, Maisonobe T, Knauss S, Ben Hadj Salem O, Hervier B, Auré K, et al. Immune checkpoint inhibitor-related myositis and myocarditis in patients with cancer. Neurology. 2018 Sep 4;91(10):e985–94.

33. Wang C, Lu Y, Chen L, Gao T, Yang Q, Zhu C, et al. Th9 cells are subjected to PD-1/PD-L1-mediated inhibition and are capable of promoting CD8 T cell expansion through IL-9R in colorectal cancer. Int Immunopharmacol. 2020 Jan;78:106019.

34. Anghileri E, Di Ianni N, Paterra R, Langella T, Zhao J, Eoli M, et al. High tumor mutational burden and T-cell activation are associated with long-term response to anti-PD1 therapy in Lynch syndrome recurrent glioblastoma patient. Cancer Immunol Immunother CII. 2021 Mar;70(3):831–42.

35. Miao L, Wei X-L, Zhao Q, Qi J, Ren C, Wu Q-N, et al. p.P476S mutation of RBPJL inhibits the efficacy of anti-PD-1 therapy in oesophageal squamous cell carcinoma by blunting T-cell responses. Clin Transl Immunol. 2020;9(9):e1172.

36. Chung AS, Mettlen M, Ganguly D, Lu T, Wang T, Brekken RA, et al. Immune Checkpoint Inhibition is Safe and Effective for Liver Cancer Prevention in a Mouse Model of Hepatocellular Carcinoma. Cancer Prev Res Phila Pa. 2020 Nov;13(11):911–22.

37. Kanaoka K, Moriizumi K, Okada H, Iwahashi K, Tsuji H, Yasuoka H, et al. Pembrolizumab-Induced Delayed-Onset Hepatitis. Case Rep Gastroenterol. 2020 Dec;14(3):586–92.

38. Sawada K, Shonaka T, Nishikawa Y, Hasegawa K, Hayashi H, Hasebe T, et al. Successful Treatment of Nivolumab-related Cholangitis with Prednisolone: A Case Report and Review of the Literature. Intern Med Tokyo Jpn. 2019 Jun 15;58(12):1747–52.
