# Supplementary material for: A Systematic Review of the Mechanisms Involved in Immune Checkpoint Inhibitors Cardiotoxicity and Challenges to Improve Clinical Safety
Source: Front Cell Dev Biol. 2022 Mar 30;10:851032. doi: 10.3389/fcell.2022.851032 (PMC9006991; doi:10.3389/fcell.2022.851032)
Supplement: Supplementary file 4 [file Table5.DOCX]

| Supplementary Table 5. Cytokine-mediated irAEs after ICIs therapies. | | | | | |
| --- | --- | --- | --- | --- | --- |
| ICI | **Source** | **IrAEs** | **Cyokine** | **PMID** | **REF** |
| Anti-CTLA-4 | P | NA | IL-4, **IFN-Y**, IL-15, | 28344869 | (1) |
| Anti-CTLA-4 | A | NA | **IFN-Y** | 11419179 | (2) |
| Anti-CTLA-4 | P | Colitis | CXCL10 | 31578309 | (3) |
| Anti-PD1 | A | NA | **IFN-γ** and IL-12 | 30552023 | (4) |
| Anti-PD1 | P | Tyroid damage | *IL-1β*, IL-2, and GM-CSF at baseline | 32086984 | (5) |
|  |  |  |  |  |  |
| Anti-PD1 | P | Rash, artralgia, colitis, pneumonitis, hipotiroidismo, hepatitis, estomatitis | IL-6, IL-10, CXCL10, IL-17A, IL-15,  *IL-1β* | 33020238 | (6) |
| Anti-PD1 | P |  |  | 32847986 | (7) |
| Anti-PD1 | A | NA | **IFNγ** | 29018057 | (8) |
| Anti-PD1 | P | NA | **IFNγ**, IL-10, IL-4, IL-12 | 33077770 | (9) |
| Anti-PD1 | P | Systemic capillary leak syndrome (SCLS) | (IL)-2, IL-6, **IFNγ** and tumor necrosis factor-α, | 32794214 | (10) |
| Anti-PD1 | P | NA | IL2 and IL21 | 30863922 | (11) |
| Nivolumab | P | NA | IL-8, IL-11 | 31562055 | (12) |
| Nivolumab | P | Vitiligo | CCL20, CCL19, CCL21, CXCL5 and CXCL1 | 31746456 | (13) |
| Nivolumab | P | NA | adiponectin; C-reactive protein (CRP); ferritin (FRTN; tumor necrosis factor receptor 2 (TNFR2); | 31721173 | (14) |
| Anti-PDL-1 | A | NA | anti-PD-L1 antibody treatment enhanced **IFN-γ** production | 24915569 | (15) |

1. Tallerico R, Cristiani CM, Staaf E, Garofalo C, Sottile R, Capone M, et al. IL-15, TIM-3 and NK cells subsets predict responsiveness to anti-CTLA-4 treatment in melanoma patients. Oncoimmunology. 2017;6(2):e1261242.

2. Paradis TJ, Floyd E, Burkwit J, Cole SH, Brunson B, Elliott E, et al. The anti-tumor activity of anti-CTLA-4 is mediated through its induction of IFN gamma. Cancer Immunol Immunother CII. 2001 May;50(3):125–33.

3. Kaesler S, Wölbing F, Kempf WE, Skabytska Y, Köberle M, Volz T, et al. Targeting tumor-resident mast cells for effective anti-melanoma immune responses. JCI Insight. 2019 Oct 3;4(19):125057.

4. Garris CS, Arlauckas SP, Kohler RH, Trefny MP, Garren S, Piot C, et al. Successful Anti-PD-1 Cancer Immunotherapy Requires T Cell-Dendritic Cell Crosstalk Involving the Cytokines IFN-γ and IL-12. Immunity. 2018 Dec 18;49(6):1148-1161.e7.

5. Kurimoto C, Inaba H, Ariyasu H, Iwakura H, Ueda Y, Uraki S, et al. Predictive and sensitive biomarkers for thyroid dysfunctions during treatment with immune-checkpoint inhibitors. Cancer Sci. 2020 May;111(5):1468–77.

6. Keegan A, Ricciuti B, Garden P, Cohen L, Nishihara R, Adeni A, et al. Plasma IL-6 changes correlate to PD-1 inhibitor responses in NSCLC. J Immunother Cancer. 2020 Oct;8(2):e000678.

7. Ng HHM, Lee RY, Goh S, Tay ISY, Lim X, Lee B, et al. Immunohistochemical scoring of CD38 in the tumor microenvironment predicts responsiveness to anti-PD-1/PD-L1 immunotherapy in hepatocellular carcinoma. J Immunother Cancer. 2020 Aug;8(2):e000987.

8. Wang J, Xie T, Wang B, William WN, Heymach JV, El-Naggar AK, et al. PD-1 Blockade Prevents the Development and Progression of Carcinogen-Induced Oral Premalignant Lesions. Cancer Prev Res Phila Pa. 2017 Dec;10(12):684–93.

9. Giunta EF, Barra G, De Falco V, Argenziano G, Napolitano S, Vitale P, et al. Baseline IFN-γ and IL-10 expression in PBMCs could predict response to PD-1 checkpoint inhibitors in advanced melanoma patients. Sci Rep. 2020 Oct 19;10(1):17626.

10. Umeda Y, Hayashi H, Sugiyama S, Aoyama Y. Systemic capillary leak syndrome triggered by anti-programmed death 1 checkpoint inhibitor in psoriasis. J Dermatol. 2020 Nov;47(11):1322–5.

11. Chat V, Ferguson R, Simpson D, Kazlow E, Lax R, Moran U, et al. Autoimmune genetic risk variants as germline biomarkers of response to melanoma immune-checkpoint inhibition. Cancer Immunol Immunother CII. 2019 Jun;68(6):897–905.

12. Agulló-Ortuño MT, Gómez-Martín Ó, Ponce S, Iglesias L, Ojeda L, Ferrer I, et al. Blood Predictive Biomarkers for Patients With Non-small-cell Lung Cancer Associated With Clinical Response to Nivolumab. Clin Lung Cancer. 2020 Jan;21(1):75–85.

13. Fujimura T, Tanita K, Sato Y, Lyu C, Kambayashi Y, Fujisawa Y, et al. Immune checkpoint inhibitor-induced vitiligo in advanced melanoma could be related to increased levels of CCL19. Br J Dermatol. 2020 May;182(5):1297–300.

14. Wang R, Shao X, Zheng J, Saci A, Qian X, Pak I, et al. A Machine-Learning Approach to Identify a Prognostic Cytokine Signature That Is Associated With Nivolumab Clearance in Patients With Advanced Melanoma. Clin Pharmacol Ther. 2020 Apr;107(4):978–87.

15. Maekawa N, Konnai S, Ikebuchi R, Okagawa T, Adachi M, Takagi S, et al. Expression of PD-L1 on canine tumor cells and enhancement of IFN-γ production from tumor-infiltrating cells by PD-L1 blockade. PloS One. 2014;9(6):e98415.
