# Supplementary material for: A Systematic Review of the Mechanisms Involved in Immune Checkpoint Inhibitors Cardiotoxicity and Challenges to Improve Clinical Safety
Source: Front Cell Dev Biol. 2022 Mar 30;10:851032. doi: 10.3389/fcell.2022.851032 (PMC9006991; doi:10.3389/fcell.2022.851032)
Supplement: Supplementary file 5 [file Table3.DOCX]

| Supplementary Table 3. Mechanisms of anti-PD-L1 T cell recruitment | | |  |  | |
| --- | --- | --- | --- | --- | --- |
| ICIs | **Source** | **Findings After treatment** | **PMID** | | **REF** |
| anti-PD-L1 | P | IL-21 is crucial for developing a robust CD8^+^ T cell response in osteosarcoma patients and is reduced by the treatment. Tfh cells presented a severe reduction in IL-21 secretion capacity as well as in proliferation capacity. | 28650673 | | (1) |
| anti-PD-L1 | P | Focal CD4^+^ T cell-rich infiltrates, CD8^+^ T cells were also present. In this patient, CD4^+^ and CD8^+^ T cells were observed in-between and inside hybrid structures. | 32300556 | | (2) |
|  |  |  |  | |  |
| Atezolizumab | P | Therapy induces a slightly elevated WBC count. Circulating lymphocyte ratio increased the frequencies of CD4^+^ and CD8^+^ T cells in peripheral blood of NSCLC patients. | 29758930 | | (3) |
| Avelumab | AM | The anti-tumor effects were more dependent on the presence of CD4^+^ than CD8^+^ T cells, as determined by *in vivo* immune cell depletions. | 26921031 | | (4) |
| Durvalumab | P | First case of durvalumab-induced Diffuse Alveolar Hemorrhage with infiltrating CD3+ and CD8+ lymphocytes in the lung interstitium, whereas CD20+ and CD4+ lymphocytes were scarcely detected. | 32774259 | | (5) |
| anti-PD-L1 | AM | Highly efficacious tumor growth inhibition and enhanced infiltration by CD4+ and CD8+ lymphocytes. | 31302474 | | (6) |

Data obtained from 4 retrieve papers of 5 analyzed. Interleukin 21 (IL-21), Tfh, white blood cells, NSCLC, P: Patients, AM: Animal model.

1. Gao W, Zhou J, Ji B. Evidence of Interleukin 21 Reduction in Osteosarcoma Patients Due to PD-1/PD-L1-Mediated Suppression of Follicular Helper T Cell Functionality. DNA Cell Biol. 2017 Sep;36(9):794–800.

2. Pringle S, van der Vegt B, Wang X, van Bakelen N, Hiltermann TJN, Spijkervet FKL, et al. Lack of Conventional Acinar Cells in Parotid Salivary Gland of Patient Taking an Anti-PD-L1 Immune Checkpoint Inhibitor. Front Oncol. 2020;10:420.

3. Zhuo M, Chen H, Zhang T, Yang X, Zhong J, Wang Y, et al. The potential predictive value of circulating immune cell ratio and tumor marker in atezolizumab treated advanced non-small cell lung cancer patients. Cancer Biomark Sect Dis Markers. 2018;22(3):467–76.

4. Vandeveer AJ, Fallon JK, Tighe R, Sabzevari H, Schlom J, Greiner JW. Systemic Immunotherapy of Non-Muscle Invasive Mouse Bladder Cancer with Avelumab, an Anti-PD-L1 Immune Checkpoint Inhibitor. Cancer Immunol Res. 2016 May;4(5):452–62.

5. Kanaoka K, Ikebe S, Ihara S, Tsuji H, Yasuoka H, Minami S. Durvalumab-Induced Diffuse Alveolar Hemorrhage: An Autopsy Case Report. Case Rep Oncol. 2020 Aug;13(2):696–701.

6. Chae YJ, Kim J, Heo H, Woo C-W, Kim S-T, Kim MJ, et al. Magnetic Resonance Colonography Enables the Efficacy Assessment of Immune Checkpoint Inhibitors in an Orthotopic Colorectal Cancer Mouse Model. Transl Oncol. 2019 Sep;12(9):1264–70.
