# Supplementary material for: A Systematic Review of the Mechanisms Involved in Immune Checkpoint Inhibitors Cardiotoxicity and Challenges to Improve Clinical Safety
Source: Front Cell Dev Biol. 2022 Mar 30;10:851032. doi: 10.3389/fcell.2022.851032 (PMC9006991; doi:10.3389/fcell.2022.851032)
Supplement: Supplementary file 6 [file Table4.DOCX]

| **Supplementary Table 4.** irAEs associated with autoantibodies-mediated ICIs. | | | | | |
| --- | --- | --- | --- | --- | --- |
| **ICIs** | **Source** | **irAE** | **Antibody specificity** | **PMID** | **REF** |
| **Anti-CTLA-4** | | | | |  |
| Ipilimumab | P | Vasculitis neuropathy | ANCA: anti-MPO and anti-PR3 | 31209477 | (1) |
| Ipilimumab | A | Thyroid dysfunction | Anti-TPO and anti-TG | 30425107 | (2) |
| Anti-CTLA-4 | P | Autoimmune hepatitis | Anti-DNA | 17508005 | (3) |
| Tremelimumab | P | Encephalomyeloradiculopathy | Anti-Ma1 and anti-Ma2 | 25661887 | (4) |
| Ipilimumab or Pembrolizumab | P | Hypophysitis | ZCCHC8 common antibodies: GGA2, RBM34, MPG, LCP1, ACVR2B,TEC,MRPS18A, RPUSD2, among others | 32671216 | (5) |
| Ipilimumab or anti-PD-1 (pembrolizumab and nivolumab) | P | Insulin-dependent diabetes | Anti-GAD65 | 30899528 | (6) |
| Ipilimumab or Nivolumab or Ipilimumab + Nivolumab | P | Inflammatory arthritis and sicca syndrome | Anti-nuclear antibodies | 27307501 | (7) |
| Ipilimumab+ Nivolumab | P | Hypoparathyroidism | Anti-CaSR | 32581059 | (8) |
| Ipilimumab+ Nivolumab | P | Retinopathy | Anti-retinal | 31246886 | (9) |
| Ipilimumab+ Nivolumab | P | Hypothyroidism | Anti-TPO, anti-TG | 29862971 | (10) |
| Ipilimumab+ Nivolumab | P | Autoimmune Encephalitis | Anti-N-methyl-D-aspartate receptor | 27271951 | (11) |
| **Anti-PD-1** | | | | |  |
| Nivolumab | P | Type 1 diabetes mellitus | Pancreatic islet-related autoantibody-negative t1dm | 30853818 | (12) |
| Nivolumab | P | Autoimmune Diabetes and Thyroiditis | Anti-GAD and anti-TPO | 28611636 | (13) |
| Nivolumab | P | Autoimmune diabetes | Anti-GAD-65, anti-TPO, insulin autoantibody | 28515940 | (14) |
| Nivolumab | P | Diabetes Mellitus | IA-2Ab antibodies | 31708539 | (15) |
| Nivolumab | P | Bullous pemphigoid | BP 180 antibodies and C-terminal domains and LAD-1 | 28865162, 31570332 | (16), (17) |
| Nivolumab | P | Cerebellar type of Hashimoto's encephalopathy | Anti-thyroid antibodies | 31462589 | (18) |
| Nivolumab | P | Autoimmune disorders | within 30 days of >1AAbs among ANAs, ENAs and ASMAs | 31289683 | (19) |
| Nivolumab | P | Hypoparathyroidism | Anti-CaSR | 30252069 | (20) |
| Nivolumab | P | Kidney injury | Anti-glomerular basement membrane | 30032835 | (21) |
| Nivolumab | P | Limbic Encephalitis | Anti-Hu | 29857970 | (22) |
| Nivolumab | P | Thrombocytopenia and hypothyroidism | Anti-TPO and anti-TG | 29260625 | (23) |
| Nivolumab | P | **Myositis /atrioventricular block** | PL-12-B (alanyl-tRNA snythetase antibodies) and anti-SRP-B | 27977496 | (24) |
| Nivolumab | P | ***Myasthenia gravis and myopathy*** | Anti-titin antibody | 31243249 | (25) |
| Nivolumab | P | ***Myasthenia gravis and myopathy*** | Acetylcholine receptor antibody | 31436031 | (26) |
| Nivolumab | P | ***Myasthenia gravis*** | Anti-AChR | 26491202 | (27) |
| Nivolumab | P | Autoimmune encephalitis | Ma2 antibodies | 32303632 | (28) |
| Nivolumab | P | Dermatomyositis | anti-transcriptional intermediary factor 1-γ (anti-TIF1-γ) antibodies | 32651759 | (29) |
| Nivolumab | P | thyroid dysfunction | antithyroglobulin antibodies | 30230649 | (30) |
| Nivolumab | P | Hypoparathyroidism | Anti- calcium-sensing receptor (CaSR) | 30252069 | (20) |
| Nivolumab | P | Encephalopathy | Hu autoantibodies | 28577954 | (31) |
| Nivolumab | P | thrombocytopenia and hypothyroidism | thyroglobulin and thyroid peroxidase antibodies | 29260625 | (23) |
| Nivolumab | P | Type 1 diabetes mellitus | Anti-glutamic acid decarboxylase antibodies (GADA) and thyroid autoantibodies | 33786278 | (32) |
| Nivolumab | P | Cerebral vasculitis | Anti-nuclear antibodies | 28642817 | (33) |
| Nivolumab | P | myasthenia gravis and rhabdomyolysis | Acetylcholine receptor binding antibodies | 26491202 | (27) |
| Nivolumab | P | Limbic Encephalitis | Anti-Hu Antibody | 29857970 | (22) |
| Pembrolizumab | P | ***Myasthenia gravis with myositis and myocarditis*** | Antibodies to striated muscle and Anti-AChR | 30528803* | (34) |
| Pembrolizumab | P | ***Myasthenia gravis and myositis*** | Anti-AChR and anti-titin | 31341124 | (35) |
| Pembrolizumab | P | ***Myositis*** | Anti-AChR, anti-striated muscle: anti-titin and anti-Kv1.4. | 31323130 | (36) |
| Pembrolizumab | P | Ocular Myasthenia Gravis | Anti-titin | 30713313 | (37) |
| Pembrolizumab | P | General autoimmunity | Anti-AChRs, anti-TG, anti-TPO, striated muscles, and mitochondrial | 32442551 | (38) |
| Pembrolizumab | P | Hypoparathyroidism | Anti-CaSR | 32112105 | (39) |
| Pembrolizumab | P | Thyroid dysfunction | Anti-TPO and/or TRAb | 27571185 | (40) |
| Pembrolizumab | P | Atypical skin toxicities | Anti-desmoplakin 1 and 2, and anti-desmoglein 1 and 3 | 27482939 | (41) |
| Pembrolizumab | P | Taxic sensory neuronopathy (SNN) | Anti-ANA, anti-SSA | 30348223 | (42) |
| Pembrolizumab | P | Bullous pemphigoid | Anti-BP180 | 28677843 | (43) |
| Pembrolizumab | P | Glomerulonephritis | Anti-neutrophil cytoplasmic | 28342816 | (44) |
| Pembrolizumab | P | Autoimmune limbic encephalitis | Anti-CASPR2 | 28284337 | (45) |
| Pembrolizumab | P | bullous pemphigoid | anti-BMZ antibodies | 33184934 | (46) |
| Pembrolizumab | P | Neuromyelitis optica spectrum disorder (NMOSD) | anti-aquaporin-4 antibody (AQP4-Ab) | 31655267 | (47) |
| Pembrolizumab | P | myositis with dropped head syndrome | anti-titin antibody | 32914675 | (48) |
| anti-PD-1 | P | Autoimmune diabetes | Anti-GAD | 31265074 | (49) |
| anti-PD-1 | P | Cerebral vasculitis/encephalitis | Vascular endothelial antibodies. | 28642817 | (33) |
| anti-PD-1 | P | Bullous pemphigoid | Anti-LAD-1 | 31474998 | (50) |
| Nivolumab, Pembrolizumab | P | ***Myasthenia Gravis and Myositis*** | Anti-striational | 32381950 | (51) |
| Nivolumab, Pembrolizumab | P | Sí | Anti-C1GALT1 | 32427353 | (52) |
| Nivolumab, Pembrolizumab | P | Thyroid dysfunction | Anti-TG and anti-TPO | 31093955 | (53) |
| Nivolumab or pembrolizumab, Ipilimumab, or Ipilimumab + Nivolumab. | P | Thyroid dysfunctions | Thyroid autoantibodies | 32086984 | (54) |
| Nivolumab, pembrolizumab, ipilimumab+nivolumab, ipilimumab+durvalumab, or atezolizumab. | P | Peripheral neuropathy | Anti-Ma2, anti-glial fibrillar acidic protein andante-contactin-associated protein-like 2 | 32312871 | (55) |
| Ipilimumab+Nivolumab | P | Cerebellar toxicity | anti-Zic4 antibody | 32943444 | (56) |
| **Anti-PD-L1** | | | | |  |
| Durvalumab | P | ***Myositis*** | Anti-3-hydroxy-3-methylglutaryl-coenzyme A reductase antibody | 32400023 | (57) |
| Durvalumab | P | Sjögren's syndrome | Anti-SSA | 32300556 | (58) |
| Durvalumab | P | Diabetic ketoacidosis | Anti-GAD | 31829972 | (59) |
| Durvalumab | P | Autoimmune diabetes with severe diabetic ketoacidosis and immune-related thyroiditis | glutamic acid decarboxylase (GAD65) | 33209634 | (60) |
| Atezolizumab. | P | Diabetic ketoacidosis | Anti-GAD | 28978581 | (61) |
| Atezolizumab | P | Type 1 diabetes | Anti‐GAD autoantibody, and anti‐islet antigen 2 autoantibody | 31282610 | (62) |
| Nivolumab, Duvalumab | P | Bullous Skin | Anti-BP180 and BP230 | 26928461 | (63) |
| Anti-PD-1 and anti-CTLA-4 | P | Encephalitis | Anti-Ma2 | 31345342 | (64) |

ANCA: Antineutrophil cytoplasmic antibodies, MPO: Myeloperoxidase, PR3: Proteinase 3, TPO: Thyroid peroxidase, TG: anti-thyroglobulin, ZCCHC8: zinc finger CCHC-type containing 8, GGA2: Golgi Associated, Gamma Adaptin Ear Containing, ARF Binding Protein 2, RBM34: RNA Binding Motif Protein 34, MPG: N-methylpurine DNA glycosylase, LCP: lymphocyte cytosolic protein 1, ACVR2B : Activin Receptor Type IIB, TEC: tec protein tyrosine kinase, MRPS18A :mitochondrial ribosomal protein S18A, RPUSD2: RNA pseudouridylate synthase domain containing 2, GAD65: glutamic acid decarboxylase 65, CaSR: constitutive androstane receptor, IA2b: Islet antigen 2b, LAD1: ladinin-1, ANA: AntiNuclear Antibodies, ENA: Extractable Nuclear Antigen, ASMAs: Anti-smooth muscle antibodies, anti-Hu: antineuronal nuclear antibody-type 1, SRP-B: signal recognition particle, AChR: Acetylcholine receptor, Kv1.4: Potassium Channel 1.4, TRAb: TSH receptor antibodies, SSA:Sjögren's-syndrome-related antigen A, BP180: Collagen XVII, CASPR2: contactin-associated protein 2, C1GALT1: Core 1 Synthase, Glycoprotein-N-Acetylgalactosamine 3-Beta-Galactosyltransferase 1, BP230: dystonin. Diseases shown in italics and bold represent those diseases to heart dysfunction.

1. Villarreal-Compagny M, Iglesias P, Marco-Hernández J, Milisenda JC, Casanova-Molla J, Hernández-Rodríguez J, et al. ANCA-associated vasculitic neuropathy during treatment with ipilimumab. Rheumatol Oxf Engl. 2020 Jan 1;59(1):251–2.

2. de Moel EC, Rozeman EA, Kapiteijn EH, Verdegaal EME, Grummels A, Bakker JA, et al. Autoantibody Development under Treatment with Immune-Checkpoint Inhibitors. Cancer Immunol Res. 2019 Jan;7(1):6–11.

3. Fevery S, Billiau AD, Sprangers B, Rutgeerts O, Lenaerts C, Goebels J, et al. CTLA-4 blockade in murine bone marrow chimeras induces a host-derived antileukemic effect without graft-versus-host disease. Leukemia. 2007 Jul;21(7):1451–9.

4. Vogrig A, Ferrari S, Tinazzi M, Manganotti P, Vattemi G, Monaco S. Anti-Ma-associated encephalomyeloradiculopathy in a patient with pleural mesothelioma. J Neurol Sci. 2015 Mar 15;350(1–2):105–6.

5. Leiter A, Gnjatic S, Fowkes M, Kim-Schulze S, Laface I, Galsky MD, et al. A COMMON PITUITARY AUTOANTIBODY IN TWO PATIENTS WITH IMMUNE CHECKPOINT INHIBITOR-MEDIATED HYPOPHYSITIS: ZCCHC8. AACE Clin Case Rep. 2020 Aug;6(4):e151–60.

6. Kotwal A, Haddox C, Block M, Kudva YC. Immune checkpoint inhibitors: an emerging cause of insulin-dependent diabetes. BMJ Open Diabetes Res Care. 2019;7(1):e000591.

7. Cappelli LC, Gutierrez AK, Baer AN, Albayda J, Manno RL, Haque U, et al. Inflammatory arthritis and sicca syndrome induced by nivolumab and ipilimumab. Ann Rheum Dis. 2017 Jan;76(1):43–50.

8. Dadu R, Rodgers TE, Trinh VA, Kemp EH, Cubb TD, Patel S, et al. Calcium-sensing receptor autoantibody-mediated hypoparathyroidism associated with immune checkpoint inhibitor therapy: diagnosis and long-term follow-up. J Immunother Cancer. 2020 Jun;8(1):e000687.

9. Elwood KF, Pulido JS, Ghafoori SD, Harper CA, Wong RW. CHOROIDAL NEOVASCULARIZATION AND CHORIORETINAL ATROPHY IN A PATIENT WITH MELANOMA-ASSOCIATED RETINOPATHY AFTER IPILIMUMAB/NIVOLUMAB COMBINATION THERAPY. Retin Cases Brief Rep. 2021 Sep 1;15(5):514–8.

10. Haissagerre M, Prey S, Lauro C, Rousset M, Georges A, Corcuff J-B. Immunotherapy-induced hypothyroidism A report of melanoma treated by ipilimumab and nivolumab. Ann Biol Clin (Paris). 2018 Jun 1;76(3):326–8.

11. Williams TJ, Benavides DR, Patrice K-A, Dalmau JO, de Ávila ALR, Le DT, et al. Association of Autoimmune Encephalitis With Combined Immune Checkpoint Inhibitor Treatment for Metastatic Cancer. JAMA Neurol. 2016 Aug 1;73(8):928–33.

12. Sakaguchi C, Ashida K, Yano S, Ohe K, Wada N, Hasuzawa N, et al. A case of nivolumab-induced acute-onset type 1 diabetes mellitus in melanoma. Curr Oncol Tor Ont. 2019 Feb;26(1):e115–8.

13. Li L, Masood A, Bari S, Yavuz S, Grosbach AB. Autoimmune Diabetes and Thyroiditis Complicating Treatment with Nivolumab. Case Rep Oncol. 2017 Apr;10(1):230–4.

14. Godwin JL, Jaggi S, Sirisena I, Sharda P, Rao AD, Mehra R, et al. Nivolumab-induced autoimmune diabetes mellitus presenting as diabetic ketoacidosis in a patient with metastatic lung cancer. J Immunother Cancer. 2017;5:40.

15. Ohara N, Kobayashi M, Ikeda Y, Hoshi T, Morita S, Kanefuji T, et al. Non-insulin-dependent Diabetes Mellitus Induced by Immune Checkpoint Inhibitor Therapy in an Insulinoma-associated Antigen-2 Autoantibody-positive Patient with Advanced Gastric Cancer. Intern Med Tokyo Jpn. 2020 Feb 15;59(4):551–6.

16. Le Naour S, Peuvrel L, Saint-Jean M, Dreno B, Quereux G. Three new cases of bullous pemphigoid during anti-PD-1 antibody therapy. J Eur Acad Dermatol Venereol JEADV. 2018 Mar;32(3):e104–6.

17. Matsui Y, Makino T, Ishii N, Hashimoto T, Shimizu T. Detection of IgG antibodies to BP180 NC16a and C-terminal domains and LAD-1 in nivolumab-associated bullous pemphigoid. Eur J Dermatol EJD. 2019 Oct 1;29(5):554–5.

18. Maetani Y, Nezu T, Ueno H, Aoki S, Hosomi N, Maruyama H. Steroid-responsive Nivolumab-induced Involuntary Movement with Anti-thyroid Antibodies. Intern Med Tokyo Jpn. 2019 Dec 15;58(24):3577–81.

19. Giannicola R, D’Arrigo G, Botta C, Agostino R, Del Medico P, Falzea AC, et al. Early blood rise in auto-antibodies to nuclear and smooth muscle antigens is predictive of prolonged survival and autoimmunity in metastatic-non-small cell lung cancer patients treated with PD-1 immune-check point blockade by nivolumab. Mol Clin Oncol. 2019 Jul;11(1):81–90.

20. Piranavan P, Li Y, Brown E, Kemp EH, Trivedi N. Immune Checkpoint Inhibitor-Induced Hypoparathyroidism Associated With Calcium-Sensing Receptor-Activating Autoantibodies. J Clin Endocrinol Metab. 2019 Feb 1;104(2):550–6.

21. Takahashi N, Tsuji K, Tamiya H, Shinohara T, Kuroda N, Takeuchi E. Goodpasture’s disease in a patient with advanced lung cancer treated with nivolumab: An autopsy case report. Lung Cancer Amst Neth. 2018 Aug;122:22–4.

22. Matsuoka H, Kimura H, Koba H, Tambo Y, Ohkura N, Hara J, et al. Nivolumab-induced Limbic Encephalitis with Anti-Hu Antibody in a Patient With Advanced Pleomorphic Carcinoma of the Lung. Clin Lung Cancer. 2018 Sep;19(5):e597–9.

23. Jotatsu T, Oda K, Yamaguchi Y, Noguchi S, Kawanami T, Kido T, et al. Immune-mediated thrombocytopenia and hypothyroidism in a lung cancer patient treated with nivolumab. Immunotherapy. 2018 Feb;10(2):85–91.

24. Behling J, Kaes J, Münzel T, Grabbe S, Loquai C. New-onset third-degree atrioventricular block because of autoimmune-induced myositis under treatment with anti-programmed cell death-1 (nivolumab) for metastatic melanoma. Melanoma Res. 2017 Apr;27(2):155–8.

25. Isami A, Uchiyama A, Shimaoka Y, Suzuki S, Kawachi I, Fujita N. [A case of anti-titin antibody positive nivolumab-related necrotizing myopathy with myasthenia gravis]. Rinsho Shinkeigaku. 2019 Jul 31;59(7):431–5.

26. Kim J-S, Nam T-S, Kim J, Kho B-G, Park C-K, Oh I-J, et al. Myasthenia gravis and myopathy after nivolumab treatment for non-small cell lung carcinoma: A case report. Thorac Cancer. 2019 Oct;10(10):2045–9.

27. Shirai T, Sano T, Kamijo F, Saito N, Miyake T, Kodaira M, et al. Acetylcholine receptor binding antibody-associated myasthenia gravis and rhabdomyolysis induced by nivolumab in a patient with melanoma. Jpn J Clin Oncol. 2016 Jan;46(1):86–8.

28. Lyons S, Joyce R, Moynagh P, O’Donnell L, Blazkova S, Counihan TJ. Autoimmune encephalitis associated with Ma2 antibodies and immune checkpoint inhibitor therapy. Pract Neurol. 2020 May;20(3):256–9.

29. Osaki M, Tachikawa R, Ohira J, Hara S, Tomii K. Anti-transcriptional intermediary factor 1-γ antibody-positive dermatomyositis induced by nivolumab for lung adenocarcinoma: A case report. Invest New Drugs. 2021 Feb;39(1):251–5.

30. Kimbara S, Fujiwara Y, Iwama S, Ohashi K, Kuchiba A, Arima H, et al. Association of antithyroglobulin antibodies with the development of thyroid dysfunction induced by nivolumab. Cancer Sci. 2018 Nov;109(11):3583–90.

31. Raskin J, Masrori P, Cant A, Snoeckx A, Hiddinga B, Kohl S, et al. Recurrent dysphasia due to nivolumab-induced encephalopathy with presence of Hu autoantibody. Lung Cancer Amst Neth. 2017 Jul;109:74–7.

32. Yamaguchi H, Miyoshi Y, Uehara Y, Fujii K, Nagata S, Obata Y, et al. Case of slowly progressive type 1 diabetes mellitus with drastically reduced insulin secretory capacity after immune checkpoint inhibitor treatment for advanced renal cell carcinoma. Diabetol Int. 2021 Apr;12(2):234–40.

33. Läubli H, Hench J, Stanczak M, Heijnen I, Papachristofilou A, Frank S, et al. Cerebral vasculitis mimicking intracranial metastatic progression of lung cancer during PD-1 blockade. J Immunother Cancer. 2017;5:46.

34. Shirai T, Kiniwa Y, Sato R, Sano T, Nakamura K, Mikoshiba Y, et al. Presence of antibodies to striated muscle and acetylcholine receptor in association with occurrence of myasthenia gravis with myositis and myocarditis in a patient with melanoma treated with an anti-programmed death 1 antibody. Eur J Cancer Oxf Engl 1990. 2019 Jan;106:193–5.

35. Noda T, Kageyama H, Miura M, Tamura T, Ito H. [A case of myasthenia gravis and myositis induced by pembrolizumab]. Rinsho Shinkeigaku. 2019 Aug 29;59(8):502–8.

36. Sekiguchi K, Hashimoto R, Noda Y, Tachibana H, Otsuka Y, Chihara N, et al. Diaphragm involvement in immune checkpoint inhibitor-related myositis. Muscle Nerve. 2019 Oct;60(4):E23–5.

37. Onda A, Miyagawa S, Takahashi N, Gochi M, Takagi M, Nishino I, et al. Pembrolizumab-induced Ocular Myasthenia Gravis with Anti-titin Antibody and Necrotizing Myopathy. Intern Med Tokyo Jpn. 2019 Jun 1;58(11):1635–8.

38. Fuentes-Antrás J, Peinado P, Guevara-Hoyer K, Del Arco CD, Sánchez-Ramón S, Aguado C. Fatal autoimmune storm after a single cycle of anti-PD-1 therapy: A case of lethal toxicity but pathological complete response in metastatic lung adenocarcinoma. Hematol Oncol Stem Cell Ther. 2020 May 15;S1658-3876(20)30098-4.

39. Lupi I, Brancatella A, Cetani F, Latrofa F, Kemp EH, Marcocci C. Activating Antibodies to The Calcium-sensing Receptor in Immunotherapy-induced Hypoparathyroidism. J Clin Endocrinol Metab. 2020 May 1;105(5):dgaa092.

40. de Filette J, Jansen Y, Schreuer M, Everaert H, Velkeniers B, Neyns B, et al. Incidence of Thyroid-Related Adverse Events in Melanoma Patients Treated With Pembrolizumab. J Clin Endocrinol Metab. 2016 Nov;101(11):4431–9.

41. Brunet-Possenti F, Mignot S, Deschamps L, Descamps V. Antiepidermis autoantibodies induced by anti-PD-1 therapy in metastatic melanoma. Melanoma Res. 2016 Oct;26(5):540–3.

42. Ghosn J, Vicino A, Michielin O, Coukos G, Kuntzer T, Obeid M. A severe case of neuro-Sjögren’s syndrome induced by pembrolizumab. J Immunother Cancer. 2018 Oct 22;6(1):110.

43. Wada N, Uchi H, Furue M. Bullous pemphigoid induced by pembrolizumab in a patient with advanced melanoma expressing collagen XVII. J Dermatol. 2017 Oct;44(10):e240–1.

44. Heo MH, Kim HK, Lee H, Ahn M-J. Antineutrophil Cytoplasmic Antibody-Associated Rapid Progressive Glomerulonephritis after Pembrolizumab Treatment in Thymic Epithelial Tumor: A Case Report. J Thorac Oncol Off Publ Int Assoc Study Lung Cancer. 2017 Aug;12(8):e103–5.

45. Brown MP, Hissaria P, Hsieh AH, Kneebone C, Vallat W. Autoimmune limbic encephalitis with anti-contactin-associated protein-like 2 antibody secondary to pembrolizumab therapy. J Neuroimmunol. 2017 Apr 15;305:16–8.

46. Sugawara A, Koga H, Abe T, Ishii N, Nakama T. Lichen planus-like lesion preceding bullous pemphigoid development after programmed cell death protein-1 inhibitor treatment. J Dermatol. 2021 Mar;48(3):401–4.

47. Shimada T, Hoshino Y, Tsunemi T, Hattori A, Nakagawa E, Yokoyama K, et al. Neuromyelitis optica spectrum disorder after treatment with pembrolizumab. Mult Scler Relat Disord. 2020 Jan;37:101447.

48. Takahashi S, Mukohara S, Hatachi S, Yamashita M, Kumagai S. A case of myositis with dropped head syndrome and anti-titin antibody positivity induced by pembrolizumab. Scand J Rheumatol. 2020 Nov;49(6):509–11.

49. Tsang VHM, McGrath RT, Clifton-Bligh RJ, Scolyer RA, Jakrot V, Guminski AD, et al. Checkpoint Inhibitor-Associated Autoimmune Diabetes Is Distinct From Type 1 Diabetes. J Clin Endocrinol Metab. 2019 Nov 1;104(11):5499–506.

50. Sadik CD, Langan EA, Grätz V, Zillikens D, Terheyden P. Checkpoint Inhibition May Trigger the Rare Variant of Anti-LAD-1 IgG-Positive, Anti-BP180 NC16A IgG-Negative Bullous Pemphigoid. Front Immunol. 2019;10:1934.

51. Suzuki S. [Myasthenia Gravis and Myositis(PD-1 Myopathy)]. Gan To Kagaku Ryoho. 2020 Feb;47(2):219–23.

52. Lin M-C, Huang M-C, Lou P-J. Anti-C1GALT1 Autoantibody Is a Novel Prognostic Biomarker for Patients With Head and Neck Cancer. The Laryngoscope. 2021 Jan;131(1):E196–202.

53. Mazarico I, Capel I, Giménez-Palop O, Albert L, Berges I, Luchtenberg F, et al. Low frequency of positive antithyroid antibodies is observed in patients with thyroid dysfunction related to immune check point inhibitors. J Endocrinol Invest. 2019 Dec;42(12):1443–50.

54. Kurimoto C, Inaba H, Ariyasu H, Iwakura H, Ueda Y, Uraki S, et al. Predictive and sensitive biomarkers for thyroid dysfunctions during treatment with immune-checkpoint inhibitors. Cancer Sci. 2020 May;111(5):1468–77.

55. Vogrig A, Muñiz-Castrillo S, Joubert B, Picard G, Rogemond V, Marchal C, et al. Central nervous system complications associated with immune checkpoint inhibitors. J Neurol Neurosurg Psychiatry. 2020 Jul;91(7):772–8.

56. Iyer SG, Khakoo NS, Aitcheson G, Perez C. Case of anti-Zic4 antibody-mediated cerebellar toxicity induced by dual checkpoint inhibition in head and neck squamous cell carcinoma. BMJ Case Rep. 2020 Sep 17;13(9):e235607.

57. von Itzstein MS, Khan S, Popat V, Lu R, Khan SA, Fattah FJ, et al. Statin Intolerance, Anti-HMGCR Antibodies, and Immune Checkpoint Inhibitor-Associated Myositis: A “Two-Hit” Autoimmune Toxicity or Clinical Predisposition? The Oncologist. 2020 Aug;25(8):e1242–5.

58. Pringle S, van der Vegt B, Wang X, van Bakelen N, Hiltermann TJN, Spijkervet FKL, et al. Lack of Conventional Acinar Cells in Parotid Salivary Gland of Patient Taking an Anti-PD-L1 Immune Checkpoint Inhibitor. Front Oncol. 2020;10:420.

59. Patel S, Chin V, Greenfield JR. Durvalumab-induced diabetic ketoacidosis followed by hypothyroidism. Endocrinol Diabetes Metab Case Rep. 2019 Dec 12;2019:EDM190098.

60. Lopes AR, Russo A, Li AY, McCusker MG, Kroopnick JM, Scilla K, et al. Development of autoimmune diabetes with severe diabetic ketoacidosis and immune-related thyroiditis secondary to durvalumab: a case report. Transl Lung Cancer Res. 2020 Oct;9(5):2149–56.

61. Way J, Drakaki A, Drexler A, Freeby M. Anti-PD-L1 therapy and the onset of diabetes mellitus with positive pancreatic autoantibodies. BMJ Case Rep. 2017 Oct 4;2017:bcr-2017-220415.

62. Honoki H, Yagi K, Kambara K, Chujo D, Shikata M, Enkaku A, et al. Anti-programmed death ligand 1 therapy-induced type 1 diabetes presenting with multiple islet-related autoantibodies. J Diabetes Investig. 2020 Jan;11(1):253–4.

63. Naidoo J, Schindler K, Querfeld C, Busam K, Cunningham J, Page DB, et al. Autoimmune Bullous Skin Disorders with Immune Checkpoint Inhibitors Targeting PD-1 and PD-L1. Cancer Immunol Res. 2016 May;4(5):383–9.

64. Du Rusquec P, Peyre A, Toulgoat F, Honnorat J, Raimbourg J. Fatal Anti-Ma2 Encephalitis Related to Treatment of Malignant Pleural Mesothelioma With a Combination of Anti-Programmed Death 1 and Anti-Cytotoxic T-Lymphocyte Associated Protein 4 Antibodies. J Thorac Oncol Off Publ Int Assoc Study Lung Cancer. 2019 Aug;14(8):e174–6.
