# Supplementary material for: A Systematic Review of the Mechanisms Involved in Immune Checkpoint Inhibitors Cardiotoxicity and Challenges to Improve Clinical Safety
Source: Front Cell Dev Biol. 2022 Mar 30;10:851032. doi: 10.3389/fcell.2022.851032 (PMC9006991; doi:10.3389/fcell.2022.851032)
Supplement: Supplementary file 7 [file Image1.PDF]

Supplementary Figure 1. Systematic review of the potential immune mechanisms underlying cardiac irAEs

Identification

PUBMED

↓

After duplicated elimination, 341 papers were analysed for T cell recruitment, 136 papers for autoantibodies development and y 665 papers for cytokine production until December 2020

↓

Screening

Title/abstract, study details and results were screening

↓

Studies were eliminated according to exclusion criteria.

↓

Eligibility

Screening

↓

76, 69, and 20 papers were assessed for eligibility for T cell recruitment, autoantibodies development and for cytokine production respectively

↓

Included

160 studies were used to determine the mechanisms of ICIs
